# Enhancing the Peroxygenase Activity of a Cofactor‐Independent Peroxyzyme by Directed Evolution Enabling Gram‐Scale Epoxide Synthesis

**DOI:** 10.1002/chem.202201651

**Published:** 2022-08-26

**Authors:** Marie‐Cathérine Sigmund, Guangcai Xu, Eleonora Grandi, Gerrit J. Poelarends

**Affiliations:** ^1^ Department of Chemical and Pharmaceutical Biology University of Groningen Antonius Deusignlaan 1 9713 AV Groningen The Netherlands

**Keywords:** cofactor-independent, directed evolution, epoxidation, peroxygenase, peroxyzyme

## Abstract

Peroxygenases selectively incorporate oxygen into organic molecules making use of the environmentally friendly oxidant H_2_O_2_ with water being the sole by‐product. These biocatalysts can provide ‘green’ routes for the synthesis of enantioenriched epoxides, which are fundamental intermediates in the production of pharmaceuticals. The peroxyzyme 4‐oxalocrotonate tautomerase (4‐OT), catalysing the epoxidation of a variety of α,β‐unsaturated aldehydes with H_2_O_2_, is outstanding because of its independence from any cost‐intensive cofactor. However, its low‐level peroxygenase activity and the decrease in the enantiomeric excess of the corresponding α,β‐epoxy‐aldehydes under preparative‐scale conditions is limiting the potential of 4‐OT. Herein we report the directed evolution of a tandem‐fused 4‐OT variant, which showed an ∼150‐fold enhanced peroxygenase activity compared to 4‐OT wild type, enabling the synthesis of α,β‐epoxy‐aldehydes in milligram‐ and gram‐scale with high enantiopurity (up to 98 % ee) and excellent conversions. This engineered cofactor‐independent peroxyzyme can provide new opportunities for the eco‐friendly and practical synthesis of enantioenriched epoxides at large scale.

## Graphical abstract

**Directed evolution** of the cofactor‐independent peroxyzyme 4‐OT yielded an efficient biocatalyst (fused 4‐OT P8a) for the enantioselective epoxidation of α,β‐unsaturated aldehydes using hydrogen peroxide as an oxidant. The optimized peroxyzyme enabled the milligram‐ and gram‐scale preparation of the desired α,β‐epoxy‐aldehydes with excellent conversions and outstanding enantiopurity, providing an attractive route towards the synthesis of valuable intermediates for the production of pharmaceuticals.

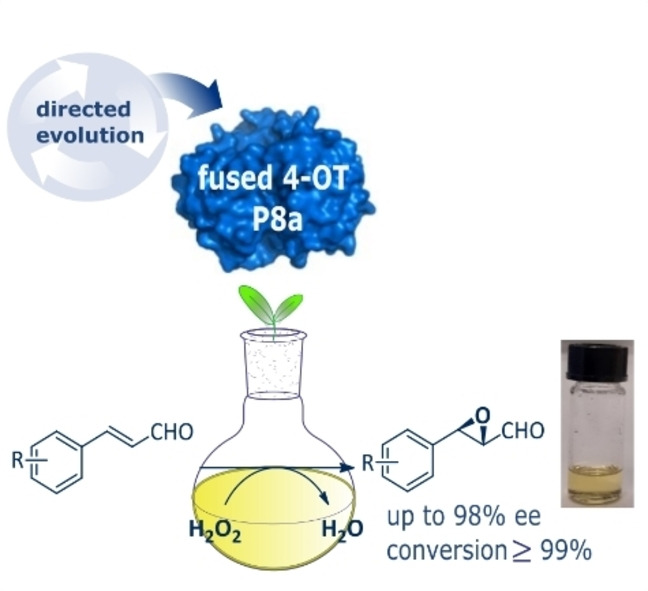

## Introduction

Epoxides, also called oxiranes, are important intermediates in the synthesis of pharmaceuticals and natural products. The high stress of the three‐membered C−O−C ring and the polarization of the carbon‐oxygen bond make epoxides intrinsically reactive compounds, which can be further derivatized by the attack of a variety of nucleophiles to yield, for example, halohydrins, diols or hydroxyl sulphides.[^1^, ^2^, ^3^] ‘Green’ routes to synthesize value‐added chemicals containing an oxirane moiety are highly desired. Peroxygenases can selectively incorporate oxygen into organic molecules to form epoxides under mild conditions making use of the environmentally friendly oxidant H_2_O_2_ with water being the sole by‐product.[^4^, ^5^] Five groups of enzymes are considered as ‘true’ peroxygenases based on the Enzyme Commission (EC) number classification, including the recently discovered unspecific peroxygenases (UPO's).^[4]^ All other enzymes, which can selectively incorporate peroxide‐derived oxygen into organic molecules but are not classified as peroxygenases, are referred to as peroxyzymes.^[5]^ Molecular oxygen can also serve as oxygen source for epoxidation reactions, which are catalysed by oxygenases like cytochrome P450s.^[6]^ H_2_O_2_ has, other than molecular oxygen, the advantage of being miscible with water and is relatively easy to handle in large‐scale applications.^[7]^ While the majority of enzymes catalysing peroxygenation reactions make use of a cofactor like haem,^[4]^ the recently reported peroxygenase‐like activity of the promiscuous 4‐oxalocrotonate tautomerase (4‐OT) is exceptional as 4‐OT is independent from any cofactor.^[8]^ To enhance its promiscuous peroxygenase activity, 4‐OT was engineered via a mutability‐landscape guided engineering approach yielding the enzyme variant 4‐OT YIA (Q4Y/M45I/F50A). Remarkably, the cofactor‐independent peroxyzyme 4‐OT YIA catalyses the enantioselective epoxidation of various substituted cinnamaldehydes, using either hydrogen peroxide or tert‐butyl peroxide as oxidant, providing access to both enantiomers of the corresponding α,β‐epoxy‐aldehydes in *syn*‐configuration with high enantiopurity of up to 98 % ee.^[8]^ The high enantioselectivity in the synthesis of the α,β‐epoxy‐aldehydes observed under analytical‐scale conditions was retained as well under preparative‐scale conditions when tert‐butyl peroxide was applied in the 4‐OT(YIA)‐catalysed epoxidation reaction. However, when H_2_O_2_ was used as an oxidant a significant reduction in the enantiomeric excess of the corresponding α,β‐epoxy‐aldehydes under preparative‐scale conditions was observed.^[8]^ Upon binding of cinnamaldehyde to the N‐terminal proline of 4‐OT YIA an iminium ion intermediate is formed, which can be attacked by H_2_O_2_ to yield the (2*S*,3*R*)‐epoxy‐aldehyde indicating a *si*‐face attack. The epoxy‐aldehyde product could be, in turn, spontaneously attacked by H_2_O_2_ to form the peroxyhydrate **S4** (Figure S24).^[8]^ It was hypothesized that the peroxyhydrate could act as an oxidant and, like the more bulky tert‐butyl peroxide, attack the iminium ion intermediate from the *re*‐face yielding the opposite (2*R*,3*S*)‐enantiomer of the α,β‐epoxy‐aldehyde.^[8]^ The hereby caused reduction in the enantiomeric excess of the corresponding epoxy‐aldehydes under preparative scale conditions is limiting the potential use of 4‐OT YIA in practical large‐scale reactions. Moreover, the epoxidation of α,β‐unsaturated aldehydes with H_2_O_2_ catalysed by 4‐OT YIA is with a conversion of 92 % in 12 h still relatively slow and requires further improvement.

In this study, we aimed at enhancing the epoxidation activity and enantioselectivity of the cofactor‐independent peroxyzyme 4‐OT by directed evolution, enabling a practical, large‐scale synthesis of versatile α,β‐epoxy‐aldehydes. The previously reported homotrimeric tandem‐fused 4‐OT, in which the C‐terminus of one monomer is fused using the flexible linker GGGAG to the N‐terminus of another monomer, proved to be an excellent template for enzyme engineering reducing the structural symmetry of the originally homohexameric 4‐OT and enhancing the available sequence space for the directed evolution process.^[9]^ Sequential rounds of random and targeted mutagenesis on the tandem‐fused enzyme variant and subsequent screening for improved peroxygenase activity were applied to optimize the promising biocatalyst 4‐OT for the practical synthesis of α,β‐epoxy‐aldehydes with high enantiopurity.

## Results and Discussion

**Scheme 1:**
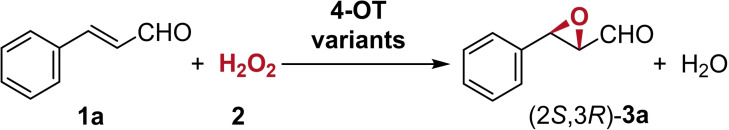
Reaction scheme of the epoxidation of cinnamaldehyde **1 a** catalysed by 4‐OT variants using H_2_O_2_ as oxidant.

The promiscuous enzyme 4‐oxalocrotonate tautomerase (4‐OT) is able to form an iminium‐ion intermediate upon the binding of cinnamaldehyde to the N‐terminal catalytic proline, which can be attacked by various nucleophiles, like nitromethane (Michael‐type addition),^[10]^ diethyl 2‐chloromalonate (cyclopropanation),^[11]^ or peroxides (peroxygenation).^[8]^ We previously reported the gene fusion and directed evolution of 4‐OT towards enhanced Michael‐type addition activity.^[9]^ Due to the similar reaction mechanisms of the Michael‐type addition and peroxygenase activity of 4‐OT, both applying iminium catalysis,^[12]^ we analysed an intermediate enzyme variant (fused 4‐OT F3a) from the above‐mentioned directed evolution trajectory for its activity towards the epoxidation of cinnamaldehyde (**1 a**) with hydrogen peroxide (**2**) to give α,β‐epoxy‐aldehyde **3 a** (Scheme 1).

**Figure 1:**
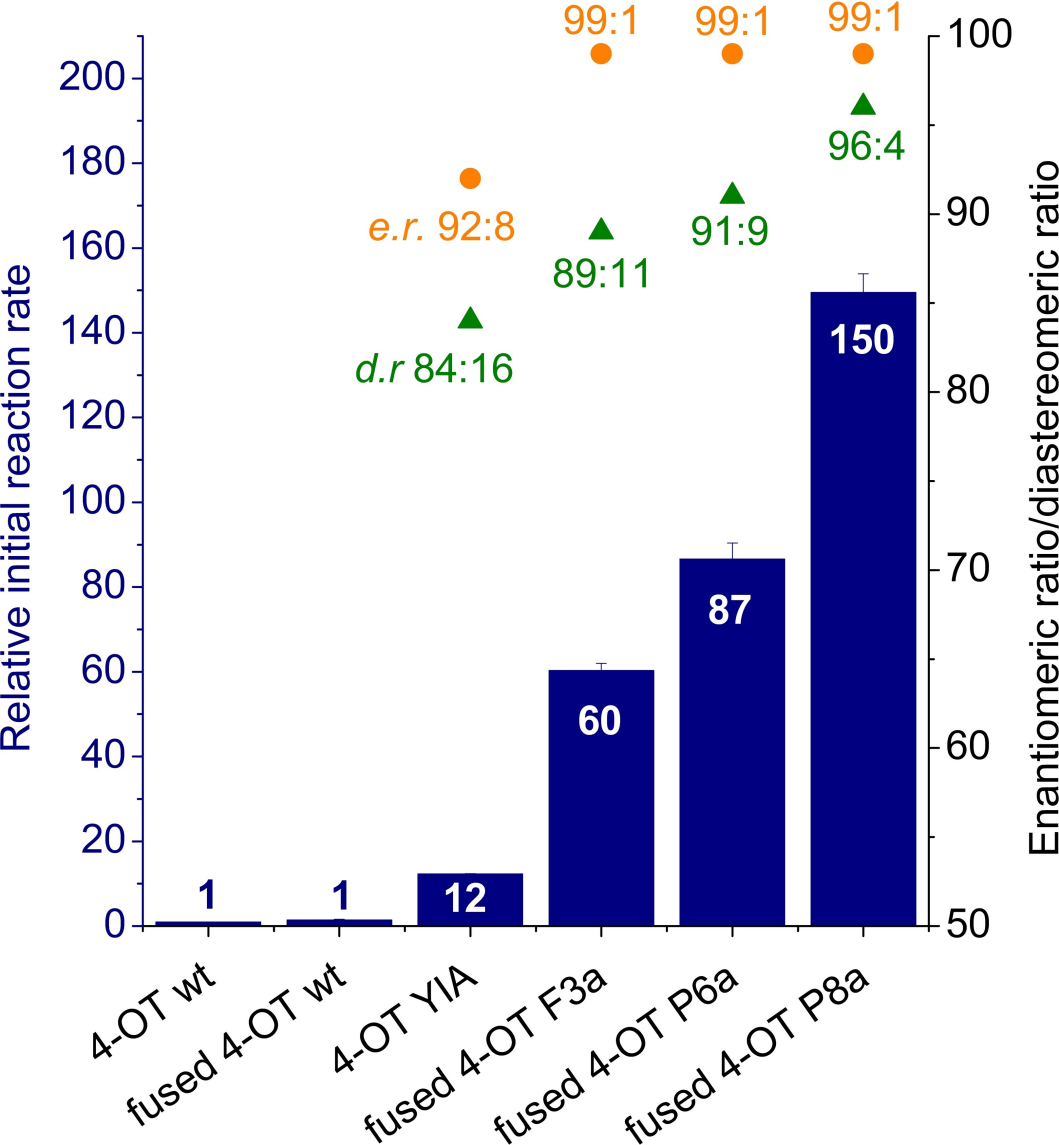
Comparison of the peroxygenase activity, and enantio‐ and diastereoselectivity of 4‐OT wild type, fused 4‐OT wild type and engineered 4‐OT variants. Bar chart showing the initial reaction rate of various 4‐OT variants (10 μM enzyme) converting 1 mM **1 a** with 50 mM H_2_O_2_ relative to the rate of the same reaction catalysed by 4‐OT wild type. The data represents the average±standard deviation from duplicate experiments. The enantiomeric ratio (e.r) and the diastereomeric ratio (d.r.; *syn*:*anti*) of the epoxide (2*S*,3*R*)‐**3 a** are represented in orange and green, respectively, for each 4‐OT variant. The product e.r. and d.r. were determined by chiral HPLC analysis.

**Figure 2:**
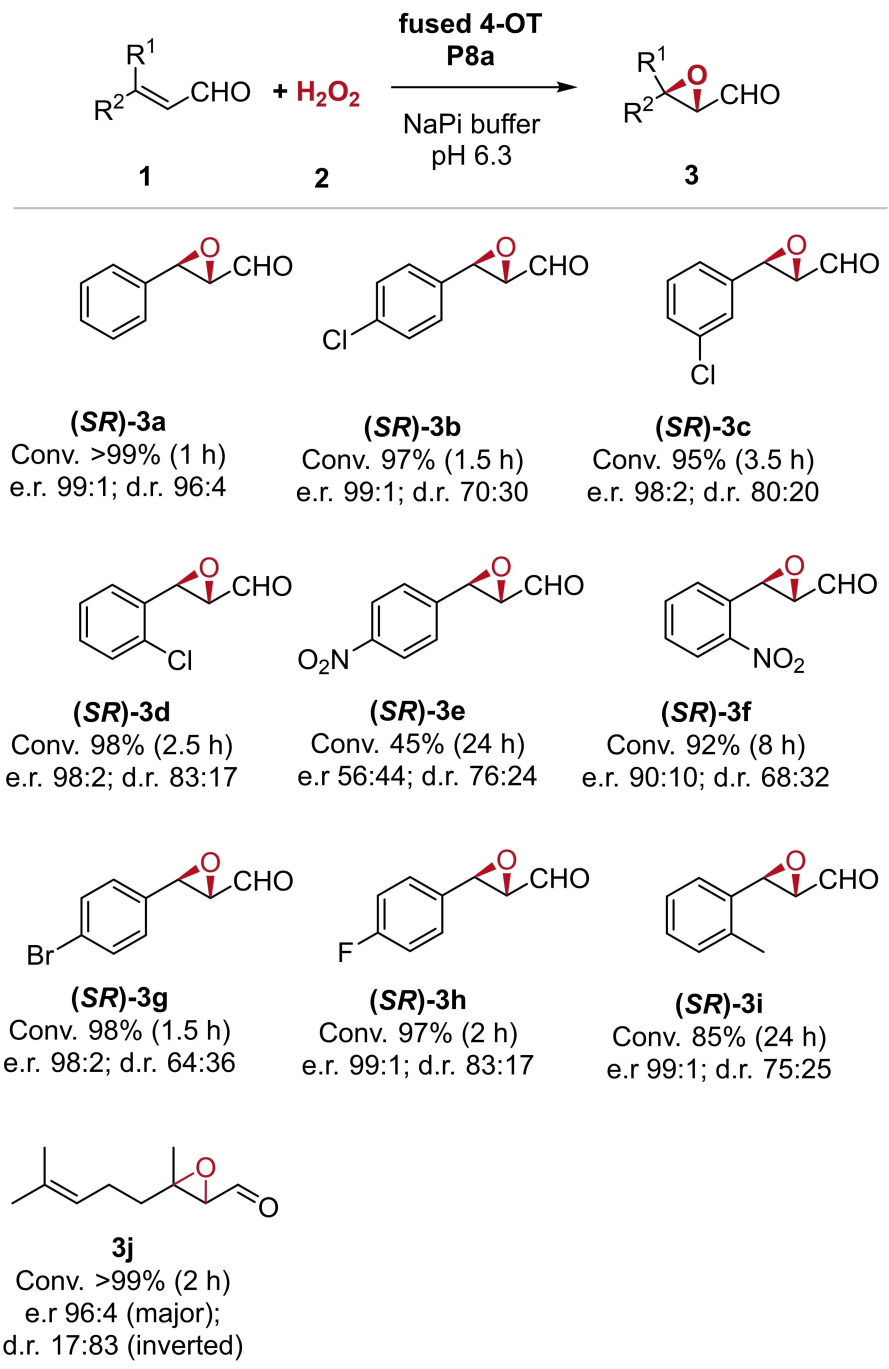
Substrate scope of fused 4‐OT P8a. Analytical scale reactions were performed with 1 mM **1**, 50 mM H_2_O_2_, and 0.14 mg mL^−1^ fused 4‐OT P8a in 20 mM sodium phosphate buffer pH 6.3 with 5 % (*v*/*v*) ethanol (MeCN for **1 e**, **1 f**, and **1 i**). Substrate conversion was determined by GC‐MS analysis. After reduction to the corresponding alcohol with NaBH_4_, the enantiomeric ratio (e.r.) and diastereomeric ratio (d.r.) of product **3** was determined by chiral HPLC analysis. The d.r. represents the ratio of syn:anti. 1j E/Z=3 : 2.

Fused 4‐OT F3a was obtained in the third round of the directed evolution trajectory (11 rounds in total) for enhanced Michael‐type addition activity and contains seven mutations on top of fused 4‐OT wild type.^[9]^ Interestingly, fused 4‐OT F3a showed an approximately 5‐times enhanced peroxygenase activity with **1 a** and **2** compared to the previously reported 4‐OT YIA and even a 60‐fold enhanced activity compared to 4‐OT wild type (Figure S25, Figure 1). Fused 4‐OT F3a was therefore used as a template for enzyme engineering to further enhance the peroxygenase activity of 4‐OT. Repeated rounds of global random mutagenesis (error‐prone PCR, epPCR),^[13]^ site‐saturation mutagenesis (SSM),^[14]^ and DNA shuffling using a PCR‐based staggered extension process (StEP)[^15^, ^16^] were applied (Table S1). In the first three rounds of directed evolution, the entire coding sequence was randomly targeted using epPCR. In round 4, SSM was applied on positions shown to be beneficial for the Michael‐type addition activity of fused 4‐OT.^[9]^ In round 5, StEP PCR was applied to shuffle beneficial mutations retrieved from the previous round of directed evolution and additionally a global random mutagenesis step was conducted. For the rapid identification of active enzyme variants within these extensive mutant libraries, the colorimetric ‘turn‐on’ probe 2‐hydroxy‐cinnamaldehyde was applied in solid‐phase pre‐screening assays.^[17]^ Subsequently, the pre‐selected enzyme variants were screened for enhanced epoxidation activity by following the depletion of **1 a** using a spectrophotometric assay. Variants which showed improved activity compared to the parental enzyme were taken along to the next round of directed evolution. After five rounds of directed evolution, the final enzyme variant (fused 4‐OT P8a) showed a remarkable 150‐fold enhancement in epoxidation activity compared to 4‐OT wild type (Figure S25, Figure 1). With a catalytic efficiency *k*_cat_/*K*_m_ of 267.9 M^−1^ s^−1^ (Table S3, Figure S2), fused 4‐OT P8a shows an about 10‐times increased catalytic efficiency compared to the previously reported variant 4‐OT YIA. To accentuate this increase in activity, fused 4‐OT P8a is able to convert more than 99 % of 1 mM **1 a** to **3 a** in 1 h (Figure 2), while it took 12 h for the previously reported 4‐OT YIA to convert 92 % of **1 a**.^[8]^ To monitor the alteration in activity and enantioselectivity during the evolution process, the enzyme variant fused 4‐OT F3a, the best mutant from round 4 of evolution fused 4‐OT P6a, and the final mutant fused 4‐OT P8a were purified and analysed (Figure S25, Figure 1). Not only the catalytic activity of the 4‐OT variants but also the enantiomeric ratio (e.r.) and the diastereomeric ratio (d.r.) of product **3 a** increased during the evolution trajectory, peaking in the epoxidation reaction catalysed by fused 4‐OT P8a with an excellent e.r of 99 : 1 and a very good d.r. of 96 : 4 (*syn*:*anti*; Figure 1). As previously observed for 4‐OT YIA, the epoxidation catalysed by fused 4‐OT P8a results in the (2*S*,3*R*)‐enantiomer of **3 a**. Notably, fused 4‐OT P8a does not only show enhanced catalytic activity and enantioselectivity, but also tolerates elevated hydrogen peroxide concentrations compared to 4‐OT YIA. While the catalytic activity of 4‐OT YIA decreases rapidly when the concentration of H_2_O_2_ exceeds 25 mM, fused 4‐OT P8a shows no decrease in epoxidation activity up to a H_2_O_2_ concentration of approx. 50 mM (Figure S3). Indeed, the inactivation of biocatalysts in the presence of elevated hydrogen peroxide concentrations is a major bottleneck in applying peroxygenases and peroxyzymes in industrial biotransformation reactions, which can be partly circumvented by the in situ generation of H_2_O_2_.[^4^, ^18^]

Next, the substrate scope of the engineered variant fused 4‐OT P8a towards a selection of α,β‐unsaturated aldehydes was explored. Fused 4‐OT P8a accepts a variety of *ortho*‐, *meta*‐, and *para*‐substituted cinnamaldehydes with conversions ranging from 92 % to >99 % within 1 h to 8 h (**1 a**–**d**, **1 f**–**h** and **1 j**, Figure 2). Fused 4‐OT P8a showed improved conversions for the substrates **1 a**–**d**, **1 f**–**h** and **1 j** compared to 4‐OT YIA (Figure S14). Furthermore, the corresponding products **3 a**–**d**, **3 f**–**h** and **3 j** of the fused 4‐OT P8a‐catalysed epoxidation reached excellent e.r. values of up to 99 : 1, surpassing the enantioselectivity of 4‐OT YIA (Table S4).

The *para*‐nitro‐substituted cinnamaldehyde **1 e** represented a rather poor substrate for both fused 4‐OT P8a and 4‐OT YIA. Although substrate **1 i** with a methyl group in the *ortho*‐position of the aromatic ring was less well converted by fused 4‐OT P8a than by 4‐OT YIA, fused 4‐OT P8a showed an improved enantio‐ and diastereoselectivity yielding an excellent product e.r. of 99 : 1. These results demonstrate that the cofactor‐independent peroxyzyme fused 4‐OT P8a is not only outstanding because of its independence from any cost‐intensive cofactor, but also because of its excellent chemo‐ and stereoselectivity in catalysing epoxidation reactions. Epoxidation reactions catalysed by the unspecific peroxygenase *Aae*UPO, for example, are hampered by poor chemoselectivity, resulting in hydroxylated side products, and show limitations in stereo‐ and regioselectivity.[^18^, ^19^] The epoxide products **3 a**–**i** of the P8a‐catalysed peroxygenation were identified to have the *syn*‐configuration with (2*S*,3*R*)‐**3** as major enantiomer, indicating the attack of the iminium‐ion intermediate by H_2_O_2_ from the *si*‐face. As previously observed for 4‐OT YIA,^[8]^ the epoxidation of the aliphatic aldehyde **1 j** catalysed by fused 4‐OT P8a results in product **3 j** in *anti*‐configuration as major diastereomer, representing an inversion of diastereoselectivity.

When performing the 4‐OT YIA‐catalysed epoxidation reaction with H_2_O_2_ as oxidant at preparative scale using 20 mM **1 a**, we observed a significant decrease in the e.r. of the corresponding epoxide product compared to analytical scale reactions with 1 mM **1 a**.^[8]^ The e.r. of **3 a** was reduced from 92 : 8 when 1 mM **1 a** was applied to 73 : 27 using 20 mM **1 a** with (2*S*,3*R*)‐**3 a** being the major enantiomer. We hypothesized that a peroxyhydrate **S4** (Figure S24), formed by the attack of H_2_O_2_ on the enzymatically produced epoxy‐aldehyde, could attack as oxidant the enzyme‐bound iminium ion intermediate from the *re*‐face similar to the more bulky tert‐butyl peroxide yielding the (2*R*,3*S*)‐enantiomer of **3 a**.^[8]^ We further hypothesized that, when preventing the attack of tert‐butyl peroxide (*re‐*face attack) but not of hydrogen peroxide (*si*‐face attack) on the substrate bound in the active site of 4‐OT, we might prevent the decline in the enantiomeric ratio of the epoxide product under preparative scale conditions using H_2_O_2_. Therefore, we tested 4‐OT variants available in our laboratory for their activity towards the two peroxides following cinnamaldehyde depletion by UV‐Vis spectroscopy (Figure S15). We found that 4‐OT IA, a variant containing the mutations M45I and F50A, showed peroxygenase activity comparable to 4‐OT YIA using H_2_O_2_ as oxidant but had only little activity when tert‐butyl peroxide was offered. As our engineering strategy is based on fused 4‐OT, we also tested the activity of fused 4‐OT IA, which contains the respective mutations of ‘unfused’ 4‐OT IA in the fused version of 4‐OT, namely M112I and F117A. We found that fused 4‐OT IA shows the same behaviour towards the two different oxidants as the ‘unfused’ 4‐OT IA. Fused 4‐OT F3a, the enzyme variant applied in this work as starting template, contains like fused 4‐OT IA the mutations M112I and F117A. We found that fused 4‐OT F3a shows, like fused 4‐OT IA, only marginal activity towards tert‐butyl peroxide as oxidant but is approximately 5‐times faster in catalysing the epoxidation of **1 a** with H_2_O_2_ compared to 4‐OT YIA. Reinforced by these observations, we tested the semi‐preparative scale synthesis of **3 a** catalysed by fused 4‐OT F3a using 20 mM **1 a** and 50 mM of H_2_O_2_ in a 1 mL‐scale reaction. As hypothesized, the e.r. of the corresponding epoxide **3 a** did not decrease in the semi‐preparative scale reactions applying fused 4‐OT F3a (Figure S16). These findings proved fused 4‐OT F3a once more to be a suitable starting template to enhance the peroxygenase activity of 4‐OT and support our above‐described hypothesis. After five rounds of directed evolution, we found that the final enzyme variant fused 4‐OT P8a shows like fused 4‐OT IA and fused 4‐OT F3a, all containing the mutations M112I and F117A, only marginal activity towards tert‐butyl peroxide. When offering H_2_O_2_ as oxidant, fused 4‐OT P8a retained its enhanced catalytic activity, excellent enantioselectivity and good diastereoselectivity observed in analytical‐scale reactions also under semi‐preparative scale conditions using 20 mM **1 a** (Figure S16).

Following, we focused on the semi‐preparative‐scale synthesis of a selection of α,β‐epoxy‐aldehydes in mg‐scale applying fused 4‐OT P8a. For this, we prepared 20 mL‐reactions containing 20 mM (**1 a**, **1 h**) or 10 mM (**1 c**, **1 d**, **1 g**) of aldehyde, 50 mM H_2_O_2_, 5 % (*v*/*v*) ethanol and 2.2 mg mL^−1^ or 1.1 mg mL^−1^ of P8a in 20 mM sodium phosphate buffer. When the reaction was finished, catalase was added to remove the residual hydrogen peroxide, which enabled us to abolish the before‐applied reduction step^[8]^ and extract the epoxy‐aldehyde instead of the alcohol. Without the removal of H_2_O_2_, the peroxide could react with the aldehyde during extraction and lead to unwanted side products. We obtained the epoxides **3 a**, **3 c**, **3 d**, **3 g**, and **3 h** in good yields of 67 % to 78 % with final amounts of 23 mg to 40 mg in very good purity (Table 1, Figure S18 to Figure S22). The conversions were excellent with reaction times reaching from 2 h to 4.5 h. Furthermore, the α,β‐epoxy‐aldehydes showed very good e.r. values up to 99 : 1, demonstrating that upscaling did not result in a significant decrease in enantioselectivity. To further demonstrate the catalytic potential of this cofactor‐independent peroxygenase, we performed the gram‐scale synthesis of **3 a** catalysed by fused 4‐OT P8a. In a reaction volume of 0.57 L with 20 mM **1 a**, 1.4 mg mL^−1^ fused 4‐OT P8a, and 50 mM H_2_O_2_, 99 % of the substrate was converted within 22 h even though the substrate was not fully soluble in the reaction buffer. The epoxy‐aldehyde (2*S*,3*R*)‐**3 a** was obtained in good yield of 69 % (1.05 g) and with excellent enantiopurity (e.r. 99 : 1) and good diastereopurity (d.r. 95 : 5) (Figure S23).

**Table 1:** Semi‐preparative‐scale synthesis of α,β‐epoxy‐aldehydes catalysed by fused 4‐OT P8a.^[a]^

**1**	R^1^	R^2^	t [h]	Conv. (yield)^[c]^ [%]	e.r.^[d]^	d.r.^[e]^	Abs. config.^[f]^
**a** ^[b]^	H	Ph	2	>99 (76)	99 : 1	96 : 4	2*S*,3*R*
**c**	H	*m*‐Cl−Ph	4.5	>99 (75)	97 : 3	87 : 13	2*S*,3*R*
**d**	H	*o*‐Cl−Ph	3.5	>99 (69)	98 : 2	80 : 20	2*S*,3*R*
**g**	H	*p*‐Br−Ph	3	>99 (78)	96 : 4	67 : 33	2*S*,3*R*
**h** ^[b]^	H	*p*‐F−Ph	3	>99 (67)	98 : 2	87 : 13	2*S*,3*R*

[a] General reaction conditions: **1** (10 mM), H_2_O_2_ (50 mM), fused 4‐OT P8a (1.1 mg mL^−1^), 5 % (*v*/*v*) ethanol in 20 mM sodium phosphate buffer pH 6.3. Reaction volume 20 mL. [b] 20 mM of **1** and 2.2 mg mL^−1^ fused 4‐OT P8a were applied. [c] The conversion was determined by GC‐MS. The yield represents the yield of diastereomeric mixture. No further product purification was required. [d] *Syn*‐diastereomer, determined by chiral HPLC analysis. [e] *Syn*:*anti*. [f] *Syn*‐diastereomer, determined using chiral HPLC and authentic standards reported by Xu et al.^[8]^

The enantioselective gram‐ and milligram‐scale synthesis of α,β‐epoxy‐aldehydes applying the H_2_O_2_‐selective fused 4‐OT P8a supports our hypothesis that, by inhibiting the *re*‐face attack by bulky peroxides like **S4** on the α,β‐unsaturated aldehydes bound to 4‐OT, we can prevent the decrease in the enantiomeric ratio of the corresponding α,β‐epoxy‐aldehydes under preparative scale conditions using H_2_O_2_ as oxidant (Figure S24).

Fused 4‐OT P8a contains 18 mutations on top of fused 4‐OT wild type. The mutations in the protein sequence of the starting template for directed evolution, namely L31D, M45L, T103I, V107I, M112I, F117A, and A124L of fused 4‐OT F3a, were conserved in the protein sequence of fused 4‐OT P8a and did not mutate further. The mutations M45Y and F50A in the ‘unfused’ 4‐OT, which correspond to positions 112 and 117 in fused 4‐OT, have previously shown to result in the opening and enlargement of a hydrophobic pocket at the back of the active site of 4‐OT which can harbour the aromatic ring of the substrate molecules.^[20]^ Mutations on the positions 45 and 50 have proven earlier to be beneficial for enhancing the Michael‐type addition,[^20^, ^21^] aldol addition,^[22]^ cyclopropanation,^[11]^ and peroxygenation^[8]^ activity of ‘unfused’ 4‐OT applying aromatic substrates. The crystal structure of the fused 4‐OT variant F11 (PDB entry 7PUO), engineered for enhanced Michael‐type addition activity, revealed that the mutations I108F, M112I, F117A, I119V and A124L shared with fused 4‐OT P8a result in the opening and reshaping of a hydrophobic pocket next to Pro‐1.^[9]^ Furthermore, the replacement of potentially oxidizable amino acids, like the surface‐exposed methionine at position 45 and arginine at position 62, which both mutated to a leucine, and the active site‐located methionine 112 that mutated to an isoleucine, could partly explain the increased tolerance of fused 4‐OT P8a towards elevated H_2_O_2_ concentrations compared to 4‐OT YIA.[^23^, ^24^] The mutation A66T in the linker connecting two 4‐OT monomers could result in slight changes in the structural organization of P8a optimizing the binding and/or orientation of the aldehyde substrate together with H_2_O_2_. The crystal structure of fused 4‐OT P8a, complexed with an α,β‐epoxy‐aldehyde product, a cinnamaldehyde substrate and/or hydrogen peroxide, could give more insight into the molecular basis of the enhanced peroxygenase activity and enantioselectivity as well as preference for the oxidant H_2_O_2_ over tert‐butyl peroxide. Such a structure could also guide forthcoming enzyme engineering endeavours of this peculiar peroxyzyme. Therefore, we have initiated studies to obtain crystal structures of fused 4‐OT P8a in complex with different ligands.

## Conclusion

In summary, we have enhanced the epoxidation activity of a cofactor‐independent peroxyzyme by directed evolution applying a tandem‐fused variant of 4‐oxalocrotonate tautomerase (4‐OT). The final enzyme variant fused 4‐OT P8a has an about 150‐times enhanced activity compared to 4‐OT wild type in catalysing the epoxidation of cinnamaldehyde with the environmentally friendly oxidant H_2_O_2_. Additionally, the improved 4‐OT variant exhibits enhanced enantioselectivity in the epoxidation of eight cinnamaldehyde derivatives and one aliphatic aldehyde, yielding α,β‐epoxy‐aldehydes with high conversions and e.r. values up to 99 : 1. The enhanced peroxygenase activity of 4‐OT further demonstrates the suitability of applying a tandem‐fused enzyme variant for protein engineering. Furthermore, we are now able to conduct the epoxidation reactions at preparative scale applying fused 4‐OT P8a and H_2_O_2_ without observing a reduction in the e.r. of the desired product. Notably, we have demonstrated the biocatalytic synthesis of five α,β‐epoxy‐aldehydes in mg‐scale and one α,β‐epoxy‐aldehyde in gram‐scale and with very good enantiopurity of up to 98 % ee, excellent conversions of ≥99 % and good yields applying the improved 4‐OT variant. The prospect of yielding versatile α,β‐epoxy‐aldehydes with high enantiopurity in gram‐scale using fused 4‐OT P8a makes a future application of this optimized cofactor‐independent peroxyzyme in enzymatic cascades attractive. Together with epoxide hydrolases or halohydrin dehalogenases environmentally friendly routes towards high‐value chiral chemicals can be provided without the need for laborious solvent‐based extraction of reaction intermediates.[^1^, ^25^, ^26^] The herein reported engineering of the promising cofactor‐independent peroxyzyme 4‐OT provides a starting point to generate a valuable biocatalyst for the environmentally friendly large‐scale synthesis of enantiopure epoxides, which are versatile synthons for production of pharmaceuticals.

## Experimental Section

**Materials**: Chemicals were purchased from Sigma‐Aldrich Chemical Co. (St. Louis, MO), TCI Europe N.V., Thermo Fisher Scientific (Geel, Belgium) or Fluorochem Co. (UK). Organic solvents were purchased from Biosolve (Valkenswaard, The Netherlands). Oligonucleotides were purchased from Eurofins Scientific (Ebersberg, Germany). Proteins were analysed by SDS‐PAGE on precast gels (NuPAGE^TM^ 12 % Bis‐Tris protein gels). High performance liquid chromatography (HPLC) was performed with a Shimadzu LC‐10AT HPLC and a Shimadzu SPD‐M20A diode array detector. GC‐MS was conducted with a Shimadzu GC‐MS‐QP2010 SE. NMR spectra were recorded on a Bruker 500 MHz machine at the Drug Design laboratory of the University of Groningen. Chemical shifts (δ) are reported in parts per million (ppm). Spectrophotometric measurements were performed on a V‐650 or V‐660 spectrophotometer from Jasco (IJsselstein, The Netherlands). Spectrophotometric measurements in 96‐well format were performed on a SPECTROstar Omega plate reader (BMG LABTECH, Isogen Life Science, de Meern, The Netherlands) in 96‐well microtiter plates (UV‐star μclear, Greiner Bio‐one). DNA was purified using a PCR purification kit (QIAquick®, QIAGEN).

## Methods

**Library construction**: Fused 4‐OT F3a served as a starting template for sequential rounds of directed evolution.^[9]^ The DNA libraries containing mutated fused 4‐OT genes in the vector pET20b were constructed by error prone PCR (epPCR) or site‐saturation mutagenesis (Table S1). To construct mutant libraries by epPCR, a PCR reaction was set up with 0.2 pg of pET20b harbouring the gene coding for fused 4‐OT, 1 U GoTaq MDx Hot start polymerase and GoTaq Flexbuffer, 1 mM dCTP and dTTP, 0.2 mM dATP and dGTP, 0.2 μM of each primer (F4OT_fw, F4OT_rv, Table S2), autoclaved Milli‐Q water, and 0.5 mM manganese chloride (PCR program: 98 °C for 60 s/40 cycles of 98 °C for 10 s, 52 °C for 30 s and 68 °C for 20 s/68 °C for 120 s). The resulting epPCR fragments and the vector pET20b were digested using NdeI and BamHI and ligated applying T4 DNA ligase. The ligation product was purified and transformed to electrocompetent *E. coli* DH5α cells. Plasmid DNA was isolated using a miniprep kit with the resulting plasmid DNA representing the mutant library. For site‐saturation mutagenesis, NNK‐mutant libraries were prepared on the positions 11, 47, 80, 85, 90, 103, 108, and 119 of fused 4‐OT P6a. The vector pET20b containing the gene coding for fused 4‐OT P6a was amplified applying the QuikChange technology. Q5 High‐Fidelity DNA Polymerase was utilized in a reaction volume of 50 μL with primers containing degenerated NNK codons (primer No. 3‐18;Table S2). The following PCR program was used: 95 °C, 1 min/18 cycles of 95 °C for 30 s, 60 °C for 1 min, 68 °C for 6 min/72 °C for 10 min. After removal of parental DNA by DpnI‐digestion, the DNA was transformed to chemocompetent *E. coli* DH5α cells and the plasmid DNA was isolated to obtain the DNA library for screening. By this, eight separate NNK‐mutant libraries were obtained. Different beneficial mutations were shuffled using the PCR‐based staggered extension process (StEP^[16]^) followed by epPCR. The genes coding for fused 4‐OT P7a–g were mixed in an equal ratio and amplified by PCR using 1 ng of template DNA and 1 U of Phusion polymerase (PCR program: 94 °C for 1 min/50 cycles of 94 °C for 30 s, 52 °C for 8 s). The obtained PCR product was used as template for epPCR. The hereby‐obtained shuffled and mutated genes were digested and ligated with cut pET20b vector DNA to obtain the DNA library.

**Library screening**: The 4‐OT mutant libraries were pre‐screened by a colorimetric agarplate assay selecting for functional 4‐OT mutant proteins. Following the growth of transformed *E. coli* BL21 (DE3) cells on LB‐agar plates (100 μg/mL ampicillin, 0.2 % *w*/*v* lactose), bacterial cells expressing functional enzyme variants were stained red by the addition of *ortho*‐hydroxy‐cinnamaldehyde applying a pre‐screening method described before.^[17]^ For each random mutagenesis library, 900 to 1100 red colonies and for site‐saturation mutagenesis libraries 90 colonies per library were picked for further activity screening by UV‐Vis spectroscopy. The single colonies were grown in 0.15 mL LB medium supplemented with 100 μg/mL ampicillin in 96‐well plates at 37 °C for 16 h. These pre‐cultures were used to inoculate 1 mL of the main culture in a 96‐deep‐well plate (growth for 3 h at 37 °C and 220 rpm, addition of 0.1 mM IPTG and growth at 18 °C for 16 h). The cells were collected by centrifugation (20 min, 4 °C, 3700 rpm) and lysed with BugBuster (100 μl per well supplemented with benzonase 1 : 1000, 20 min, 300 rpm, RT). After centrifugation (30 min, 4 °C, 3700 rpm), 20 μL of the supernatant was pipetted into each well of a 96‐well UV‐star microtiter plate. To initiate the screening, 80 μL of the reaction mix was added to each well yielding final concentrations of 0.25 mM cinnamaldehyde, 5 % (*v*/*v*) ethanol and 50 mM H_2_O_2_ in 20 mM sodium phosphate buffer (pH 6.3). The rate of cinnamaldehyde depletion was followed at 290 nm in a plate reader.

**Enzyme expression and purification**: The fused 4‐OT mutants were expressed and purified as described before with some modifications.^[27]^ A single colony of transformed chemocompetent *E. coli* BL21 (DE3) cells was used to inoculate 4 mL of LB medium (pre‐culture; grown at 37 °C at 220 rpm for 16 h). LB medium (1 L containing 100 μg/mL ampicillin) was inoculated with the pre‐culture and cells were grown at 37 °C and 220 rpm until an OD_600_ of 0.7. Next, 0.75 mM IPTG was added and the culture was grown at 20 °C for 24 h. The cells were harvested by centrifugation at 8,000 rpm for 10 min at 4 °C and stored at −20 °C until further use. The purified enzymes were analysed by SDS‐PAGE (Figure S1).

**Peroxygenase activity assay and steady state kinetic analysis**: The peroxygenase activity of 4‐OT variants was analysed by UV‐Vis spectroscopy following the depletion of **1 a** at 290 nm (ϵ=23,598 M^−1^ cm^−1^). The enzymatic assay contained 1 mM cinnamaldehyde, 50 mM H_2_O_2_, 10 μM enzyme, and 5 % (*v*/*v*) ethanol in 20 mM sodium phosphate buffer (pH 6.3). The absorption at 290 nm was followed for 1 h in 5 min intervals in 1 mm quartz cuvettes at 22 °C. The initial rates of cinnamaldehyde depletion in the first 20 min were used to compare the peroxygenase activity of the (fused) 4‐OT wild type and variants. To determine apparent steady‐state parameters *k*_cat,app_ and K_M,app_, the peroxygenation rate at different cinnamaldehyde concentrations was monitored for 4‐OT YIA and fused 4‐OT P8a (Table S3). The enzymatic assay was conducted in triplicates as described above with 0.07 mg mL^−1^ enzyme. The initial rates were fitted by non‐linear regression to the Michaelis‐Menten equation v_0_/[E]_0_=*k*_cat_[S]/(K_M_+[S]) (v_0_=initial velocity, [E]_0_=enzyme concentration, [S]=substrate concentration) using SigmaPlot® (version 14, Systat Software Inc; Figure S2).

**Substrate scope analysis of fused 4‐OT P8a (analytical scale)**: The reaction mixture consisted of 1 mM aldehyde **1 a**–**j**, 50 mM H_2_O_2_, 5 % (*v*/*v*) ethanol, and 0.14 mg mL^−1^ fused 4‐OT P8a in 20 mM sodium phosphate buffer at pH 6.3 in a final volume of 1 mL. For the substrates **1 e**, **1 f**, and **1 i** MeCN instead of EtOH was applied as co‐solvent. After completion of the reaction followed by UV‐Vis spectroscopy, 300 μL were used for extraction with ethylactetate for GC‐MS analysis to determine substrate conversion. The residual amount was reduced with sodium borohydride to the corresponding alcohol for chiral HPLC analysis (Figure S4 to Figure S13). The chiral HPLC analysis was conducted as described before.^[8]^ For the epoxides **3 a**–**c**, **3 e**, **3 g**, **3 h**, and **3 j** a Daicel CHIRALPAK® AD‐RH column (150×4.6 mm, 5 μm) was used. For the epoxides **3 d**, **3 f**, and **3 i** a CHIRALPAK® ID column (150×4.6 mm, 5 μm) was applied. An isocratic flow of 1 mL min^−1^ of MeCN in H_2_O was applied with a column temperature of 25 °C. The absolute stereochemistry of the *syn*‐diastereomers was identified by comparing with racemic reference compounds previously prepared in our laboratory and earlier reported chiral reference compounds.^[8]^ The racemic and chiral standards of the *ortho*‐NO_2_‐epoxy alcohol were prepared according to Xu et al.^[8]^ The absolute stereochemistry of the *anti*‐diastereomers was not identified.

**Preparative‐scale synthesis in milligram‐ and gram‐scale**: For the preparative‐scale synthesis in mg‐scale, a reaction mixture was set up containing 10 mM cinnamaldehyde derivative, 50 mM H_2_O_2_, 5 % (*v*/*v*) ethanol and 1.1 mg mL^−1^ of fused 4‐OT P8a in a final volume of 20 mL in 20 mM sodium phosphate buffer (pH 6.3). The reaction was incubated in a round‐bottom flask under stirring at RT for approx. 3 h. For the substrates **1 a** and **1 h** the substrate concentration was increased to 20 mM and 2.2 mg mL^−1^ of fused 4‐OT P8a was applied. After completion of the reaction, followed by UV‐Vis spectroscopy, residual H_2_O_2_ was removed by the addition of catalase. Following, the epoxide was extracted with 30 mL of ethylactetate and the solvent was evaporated to provide the crude product. For the preparative‐scale synthesis in gram‐scale, a reaction mixture was set up containing 10 mM cinnamaldehyde, 50 mM H_2_O_2_, 5 % (*v*/*v*) ethanol and 1.4 mg mL^−1^ of fused 4‐OT P8a in a final volume of 568 mL in 20 mM sodium phosphate buffer (pH 6.3). The reaction was incubated in an Erlenmeyer flask under shaking at 150 rpm at RT for 24 h. After the removal of residual H_2_O_2_ by catalase, the epoxide was extracted with 2×300 mL of ethylacetate and the solvent was evaporated to provide the crude product. The crude product was analysed by ^1^H NMR for product confirmation and to determine the conversion. After reduction with sodium borohydride to the corresponding alcohol, chiral HPLC analysis was applied (Figure S17 to Figure S23).

## Conflict of interest

The authors declare no conflict of interest.

1

## Supporting information

As a service to our authors and readers, this journal provides supporting information supplied by the authors. Such materials are peer reviewed and may be re‐organized for online delivery, but are not copy‐edited or typeset. Technical support issues arising from supporting information (other than missing files) should be addressed to the authors.

Supporting Information

## Data Availability

The data that support the findings of this study are available in the supplementary material of this article.
