# Supplementary material for: Enhancing the Peroxygenase Activity of a Cofactor‐Independent Peroxyzyme by Directed Evolution Enabling Gram‐Scale Epoxide Synthesis
Source: Chemistry. 2022 Aug 26;28(59):e202201651. doi: 10.1002/chem.202201651 (PMC9804992; doi:10.1002/chem.202201651)
Supplement: Supplementary file 1 — Supporting Information [file CHEM-28-0-s001.pdf]

Chemistry–A European Journal

Supporting Information

Enhancing the Peroxygenase Activity of a Cofactor-Independent Peroxyzyme by Directed Evolution Enabling Gram-Scale Epoxide Synthesis

Marie-Cathérine Sigmund, Guangcai Xu, Eleonora Grandi, and Gerrit J. Poelarends*

Table of Contents

Overview of the directed evolution trajectory 1
DNA and protein sequences of fused 4-OT F3a and fused 4-OT P8a........ 3
SDS PAGE analysis of purified 4-OT variants........ 4
Steady-state kinetic analysis 4
Influence of the hydrogen peroxide concentration on the epoxidation activity of fused 4-OT P8a and 4-OT YIA 6
HPLC chromatograms of analytical-scale epoxidation reactions catalysed by fused 4-OT P8a 7
Substrate scope comparison of fused 4-OT P8a and 4-OT YIA 13
Comparison of the peroxygenase activity of different 4-OT variants using either hydrogen peroxide or tert-butyl peroxide as nucleophile 15
Comparison of the enantioselectivity of different 4-OT mutants catalysing epoxidation reactions at semi-preparative scale 16
Preparative-scale synthesis applying fused 4-OT P8a........ 17
Product **3a**........ 18
Product **3c**........ 19
Product **3d** 20
Product **3g**........ 21
Product **3h** 22
Gram-scale synthesis of product **3a** 23
Supporting references........ 25

Overview of the directed evolution trajectory

Table S1 Overview of the directed evolution of fused 4-OT towards enhanced peroxygenation activity.

The mutations shown represent the mutations on top of fused 4-OT F3a. Fused 4-OT F3a contains the following mutations on top of fused 4-OT wild type, a tandem-fused variant of 4-OT in which the C-terminus of one monomer is linked to the N-terminus of a second monomer by a flexible linker (GGGAG): L31D, M45L, T103I, V107I, M112I, F117A, A124L. In red, newly acquired mutations in each round of directed evolution are highlighted. The mutant proteins P6a and P8a have been purified and analysed further. The positions 11, 47, 80, 85, 90, 103, 108, 119 chosen for the NNK library on top of

P6a were based on mutated positions of the directed evolution of fused 4-OT for improved Michael-type addition activity.[1]

Round	Starting mutant(s)	Method	Output	Mutations
**1**	F3a	epPCR	P4a	T36S, R62L
			P4b	T36S
**2**	P4a, P4b	epPCR	P5a	S36P, R62L, L75I
			P5b	T36S, R62L, E76G
			P5c	T36S, F50L, R62L
**3**	P5a, P5b, P5c	epPCR	**P6a**	K16R, T36S, F50L, R62L
			P6b	S36P, L75I
**4**	P6a, P6b	NNK library	P7a	R11V/E, K16R, T36S, F50L, R62L
			P7b	K47T/A, K16R, T36S, F50L, R62L
			P7c	D80P, K16R, T36S, F50L, R62L
			P7d	T85 F/R/D/C, K16R, T36S, F50L, R62L
			P7e	V90T, K16R, T36S, F50L, R62L
			P7f	I103V/K, K16R, T36S, F50L, R62L
			P7g	I119N, K16R, T36S, F50L, R62L
**5**	P7a - g	StEP and epPCR	**P8a**	R11H, K16R, T36S, F50L, R62L, A66T, I72V, D80G, V90T, I108F, I119V

Table S2 Primers for library construction.

No.	Name	DNA sequence
1	F4OT_fw	CTTTAAGAAGGAGATATACATATGCCG
2	F4OT_rv	CTCGAATTCGGATCCTTA
3	MCS_f4OT_11NNK_fw	CATATTCTGGAAGGT**NNK**AGTGATGAGCAGAGAGAAAC
4	MCS_f4OT_11NNK_rv	GGTTTCTCTCTGCTCATCACT**MNN**ACCTTCCAGAATATG
5	MCS_f4OT_47NNK_fw	CCGAACTGGCA**NNK**GGTCATCTTGGCATTGGTG
6	MCS_f4OT_47NNK_rv	CCAATGCCAAGATGACC**MNN**TGCCAGTTCGGTAATAATCACACG
7	MCS_f4OT_80NNK_fw	GAAGGCCGTTCG**NNK**GAGCAAAAAGAGACACTGATCCGCGAAG
8	MCS_f4OT_80NNK_rv	CGCGGATCAGTGTCTCTTTTTGCTC**MNN**CGAACGGCCTTC
9	MCS_f4OT_85NNK_fw	GGCCGTTCGGATGAGCAAAAAGAG**NNK**CTGATCCGCGAAG
10	MCS_f4OT_85NNK_rv	CTTCGCGGATCAG**MNN**CTCTTTTTGCTCATCCGAACGGCCTTC
11	MCS_f4OT_90NNK_fw	CACTGATCCGCGAA**NNK**TCAGAAGCAATTTCAC
12	MCS_f4OT_90NNK_rv	CGTGAAATTGCTTCTGA**MNN**TTCGCGGATCAGTGTC
13	MCS_f4OT_103NNK_fw	CACTGGATGCCCCTCTG**NNK**AGTGTGCGCATTATTATCACAG
14	MCS_f4OT_103NNK_rv	GTGATAATAATGCGCACACT**MNN**CAGAGGGGCATCCAG
15	MCS_f4OT_108NNK_fw	CCCTCTGATAAGTGTGCGCATT**NNK**ATCACAGAGATTGC
16	MCS_f4OT_108NNK_rv	GGCAATCTCTGTGAT**MNN**AATGCGCACACTTATCAGAGGGGCATC
17	MCS_f4OT_119NNK_fw	ACGCGGGT**NNK**GGTGGCGAGCTGTTATC

18	MCS_f4OT_119NNK_rv	ATAACAGCTCGCCACC**MNN**ACCCGCGTG

DNA and protein sequences of fused 4-OT F3a and fused 4-OT P8a

Fused 4-OT F3a (L31D, M45L, linker G63/G64/G65/A66/G67, T103I, V107I, M112I, F117A, A124L)

> Fused 4-OT F3a DNA

ATGCCGATTGCGCAGATTCATATTCTGGAAGGTCGTAGTGATGAGCAGAAAGAAACCCTGATTCGTG
AAGTTAGCGAAGCAATTAGCCGTAGCGATGATGCACCGCTGACCAGCGTTCGTGTGATTATTACCGA
ACTGGCAAAAGGTCATTTTGGCATTGGTGGTGAACTGGCAAGCAAAGTTCGTCGCGGTGGTGGTGC
AGGTCCGATCGCACAGATCCATATTTTAGAAGGCCGTTCAGATGAGCAAAAAGAGACACTGATCCGC
GAAGTTTCAGAAGCAATTTCACGTTCACTGGATGCCCCTCTGATAAGTGTGCGCATTATTATCACAGA
GATTGCCAAAGGCCACGCGGGTATCGGTGGCGAGCTGTTATCAAAAGTGCGTCGTTAA

> Fused 4-OT F3a protein

PIAQIHILEGRSDEQKETLIREVSEAISRS**D**DAPLTSVRVIITE**L**AKGHFGIGGELASKVRR**GGGAG**PIAQIHIL
EGRSDEQKETLIREVSEAISRSLDAPL**I**SVR**I**IITE**I**AKGH**A**GIGGEL**L**SKVRR

Fused 4-OT P8a (R11H, K16R, L31D, T36S, M45L, F50L, R62L, linker G63/G64/G65/A66T/G67, I72V, D80G, V90T, T103I, V107I, I108F, M112I, F117A, I119V, A124L)

> Fused 4-OT P8a DNA

ATGCCGATTGCGCAGATTCATATTCTGGAAGGTCATAGTGATGAGCAGAGAGAAACCCTGATTCGTG
AAGTTAGCGAAGCAATTAGCCGTAGCGATGATGCACCGCTGTCCAGCGTTCGTGTGATTATTACCGA
ACTGGCAAAAGGTCATCTTGGCATTGGTGGTGAACTGGCAAGCAAAGTTCGTCTCGGTGGTGGTAC
AGGTCCGATCGCACAGGTCCATATTTTAGAGGGCCGTTCAGGTGAGCAAAAAGAGACACTGATCCG
CGAAACGTCAGAAGCAATTTCACGTTCACTGGATGCCCCTCTGATAAGTGTGCGCATTTTCATCACAG
AGATTGCCAAAGGCCACGCGGGTGTCGGTGGCGAGCTGTTATCAAAAGTGCGTCGTTAA

> Fused 4-OT P8a protein

PIAQIHILEG**H**SDEQ**R**ETLIREVSEAISRS**D**DAPL**S**SVRVIITE**L**AKGH**L**GIGGELASKVR**LGGGTG**PIAQ**V**HIL
EGRS**G**EQKETLIRE**T**SEAISRSLDAPL**I**SVR**IF**ITE**I**AKGH**A**G**V**GGEL**L**SKVRR

SDS PAGE analysis of purified 4-OT variants

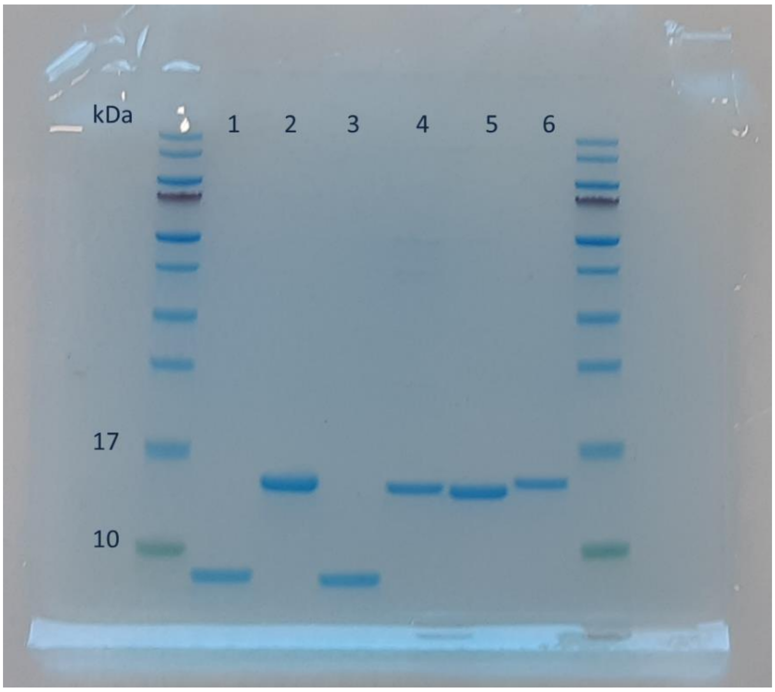

Figure S1 SDS PAGE analysis of purified 4-OT wild type and 4-OT variants.
4-OT wild type (lane 1), fused 4-OT wild type (lane 2), 4-OT YIA (lane 3), fused 4-OT F3a (lane 4), fused 4-OT P6a (lane 5), fused 4-OT P8a (lane 6). Marker: PageRuler Prestained Protein Ladder.

Steady-state kinetic analysis

Table S3 Apparent kinetic parameters of the epoxidation of cinnamaldehyde catalysed by 4-OT variants with 50 mM H_2O_2 at pH 6.3.
The enzymatic assay was conducted in 20 mM sodium phosphate buffer (pH 6.3) with 0.07 mg mL^{-1} fused 4-OT P8a or 4-OT YIA, 50 mM H_2O_2 and varying cinnamaldehyde concentrations at 22°C. The data represents the average ± standard deviation from triplicate experiments.

Enzyme	k_{cat} [s^{-1}]	K_m [mM]	k_{cat}/K_m [M^{-1} s^{-1}]
**4-OT YIA**	**0.02** ± 0.001	**0.677** ± 0.11	**28.1**
**Fused 4-OT P8a**	**0.12** ± 0.004	**0.448** ± 0.04	**267.9**

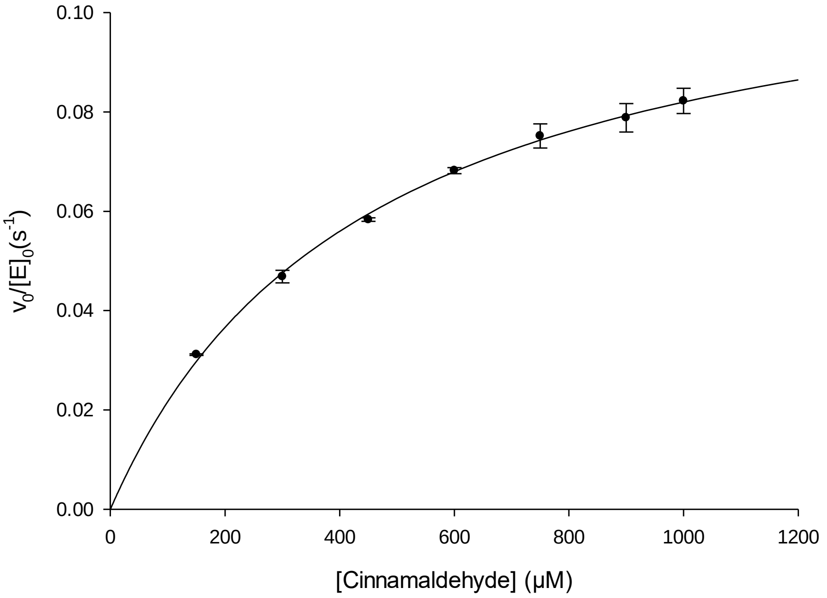

A

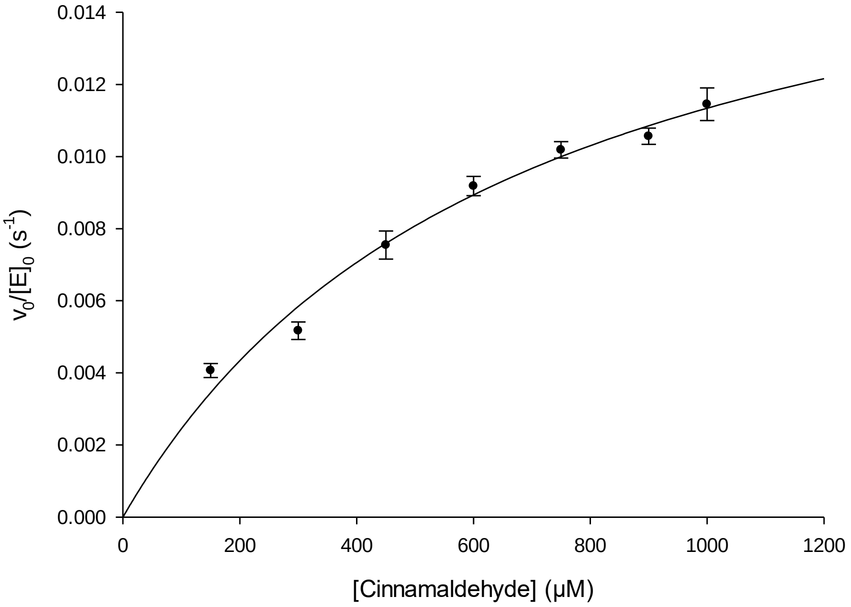

B

Figure S2 Michaelis-Menten plot for the epoxidation of cinnamaldehyde with H_2O_2 catalysed by fused 4-OT P8a or 4-OT YIA.

The enzymatic assay was conducted in 20 mM sodium phosphate buffer (pH 6.3) with 0.07 mg mL^{-1} fused 4-OT P8a (**A**) or 4-OT YIA (**B**), 50 mM H_2O_2 and varying cinnamaldehyde concentrations at 22°C. The data represents the average ± standard deviation from triplicate experiments.
(A) R^2 = 0.98, (B) R^2 = 0.95.

Influence of the hydrogen peroxide concentration on the epoxidation activity of fused 4-OT P8a and 4-OT YIA

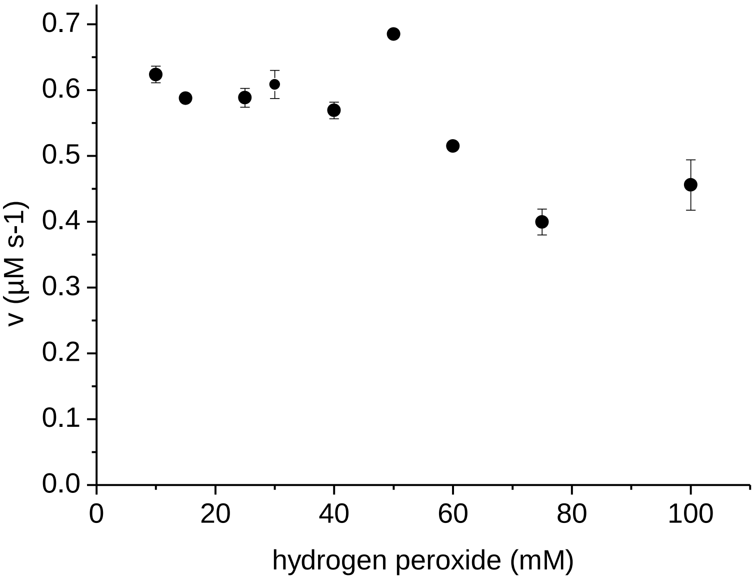

A

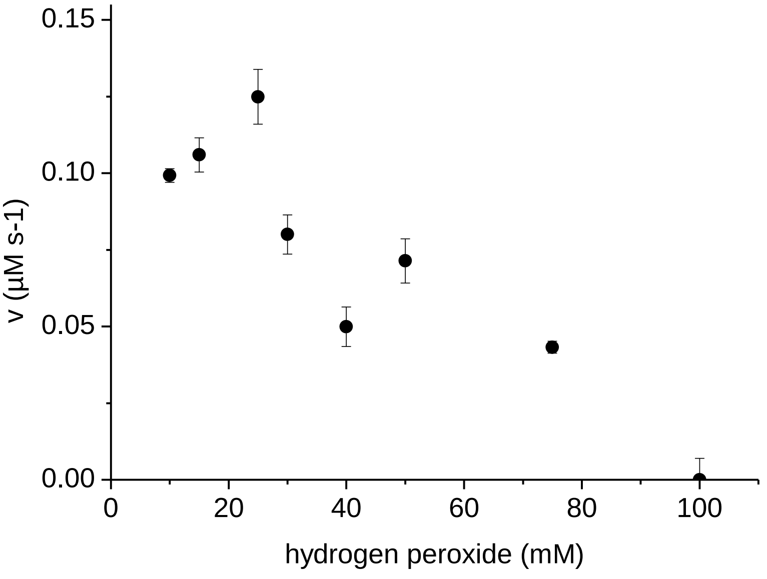

B

Figure S3 Influence of different hydrogen peroxide concentrations on the epoxidation of cinnamaldehyde catalysed by fused 4-OT P8a or 4-OT YIA.

The initial epoxidation rate in µM s^{-1} was determined by UV-Vis spectroscopy following the depletion of 1 mM **1a** in the presence of 10 µM fused 4-OT P8a (**A**) or 4-OT YIA (**B**) and varying concentrations of H_2O_2 ranging from 10 to 100 mM. The initial epoxidation rate was determined in the first 10 min of substrate depletion and represents the average ± standard deviation from duplicate experiments. The data was corrected by subtracting the rate of the background reaction when no enzyme was present.

HPLC chromatograms of analytical-scale epoxidation reactions catalysed by fused 4-OT P8a

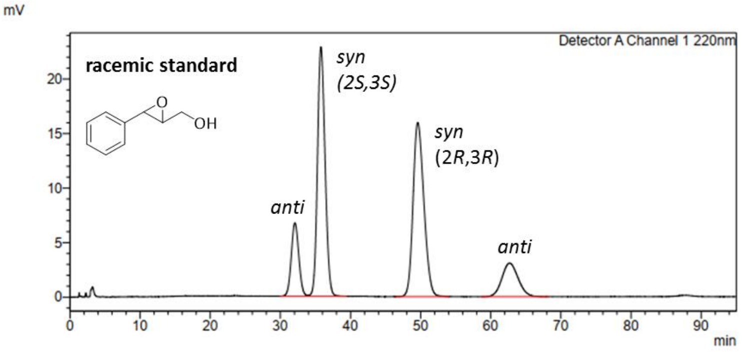

Peak#	Ret. Time	Area	Area%
1	32,071	502042	11,333
2	35,781	1716684	38,751
3	49,591	1713477	38,678
4	62,614	497885	11,239
Total		4430088	100,000

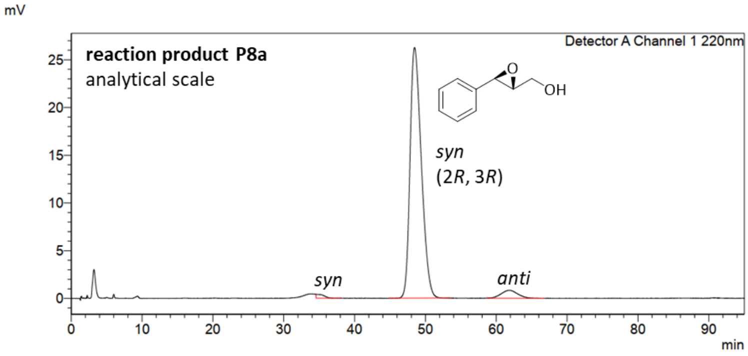

Peak#	Ret. Time	Area	Area%
1	34,597	31400	1,023
2	48,466	2903462	94,591
3	61,824	134640	4,386
Total		3069502	100,000

e.r. **99:1**

d.r. **96:4**

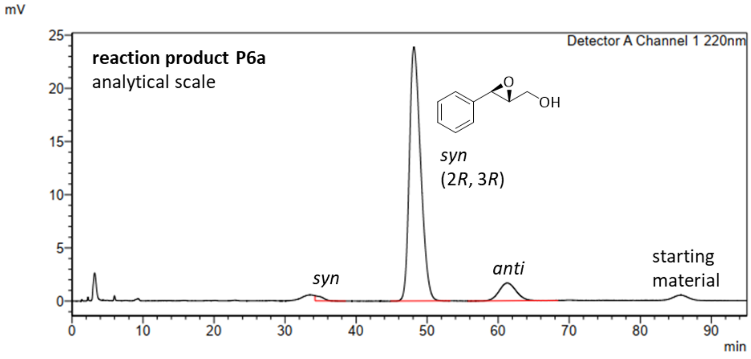

Peak#	Ret. Time	Area	Area%
1	34,248	36445	1,234
2	48,143	2638355	89,352
3	61,287	277963	9,414
Total		2952763	100,000

e.r. **99:1**

d.r. **91:9**

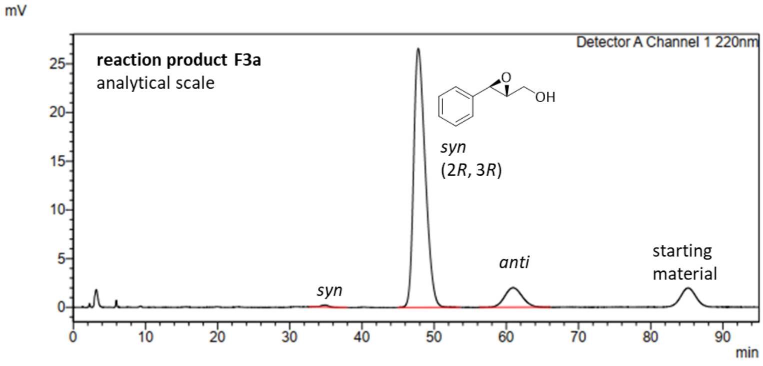

Peak#	Ret. Time	Area	Area%
1	35,009	67770	0,869
2	47,309	6842405	87,788
3	61,104	884062	11,343
Total		7794236	100,000

e.r. **99:1**

d.r. **89:11**

Figure S4 Chiral HPLC chromatograms of the alcohol derivative of product 3a obtained from enzymatic reactions at analytical scale.
Shown is the racemic standard (top), followed by the reaction products obtained from conversion by fused 4-OT P8a, P6a, or F3a.

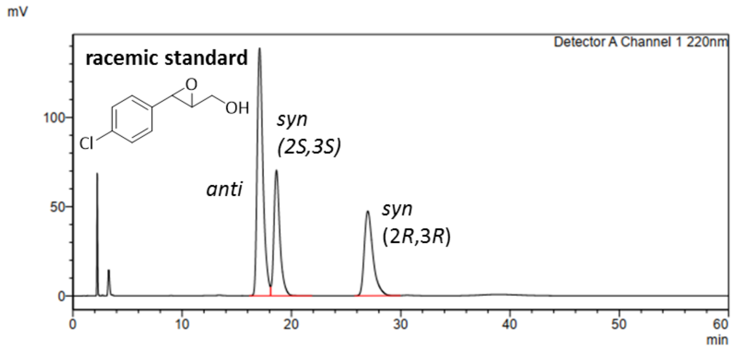

Detector A Channel 1 220nm

Peak#	Ret. Time	Area	Area%
1	17,087	5159308	49,478
2	18,629	2656198	25,473
3	26,990	2612039	25,049
Total		10427545	100,000

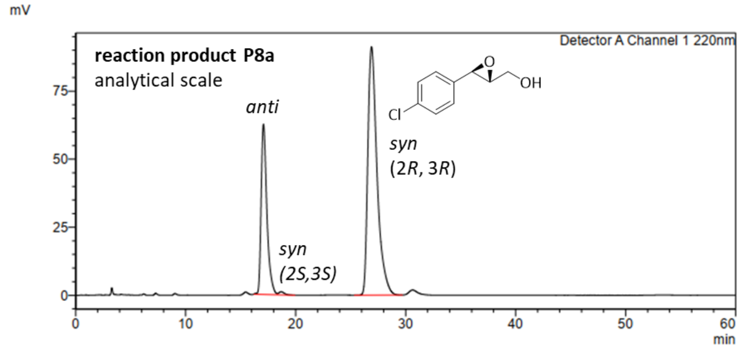

Detector A Channel 1 220nm

Peak#	Ret. Time	Area	Area%
1	17,076	2260691	29,669
2	18,680	40149	0,527
3	26,897	5318883	69,804
Total		7619723	100,000

***e.r.* 99:1**

***d.r.* 70:30**

Figure S5 Chiral HPLC chromatogram of the alcohol derivative of product 3b obtained from enzymatic reaction at analytical scale.

Shown is the racemic standard (top), and the reaction product obtained from conversion by fused 4-OT P8a (bottom).

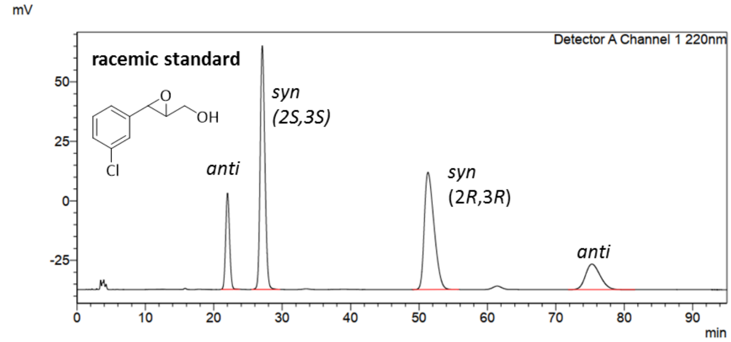

Detector A Channel 1 220nm

Peak#	Ret. Time	Area	Area%
1	22,008	1596341	12,272
2	27,087	4922570	37,844
3	51,316	4882705	37,537
4	75,301	1606049	12,347
Total		13007665	100,000

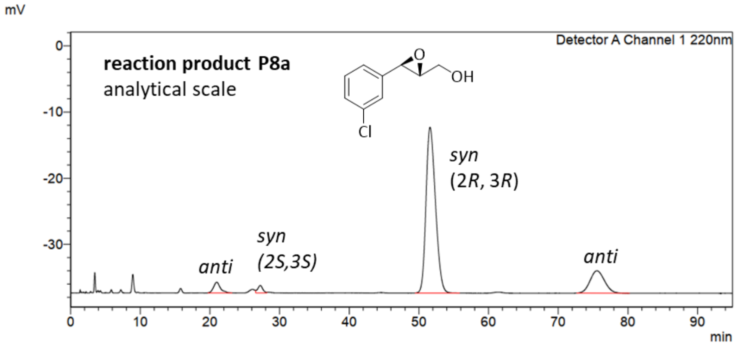

Detector A Channel 1 220nm

Peak#	Ret. Time	Area	Area%
1	20,971	111321	3,681
2	27,222	56604	1,872
3	51,601	2365219	78,216
4	75,538	490800	16,230
Total		3023944	100,000

***e.r.* 98:2**

***d.r.* 80:20**

Figure S6 Chiral HPLC chromatogram of the alcohol derivative of product 3c obtained from enzymatic reaction at analytical scale.

Shown is the racemic standard (top), and the reaction product obtained from conversion by fused 4-OT P8a (bottom).

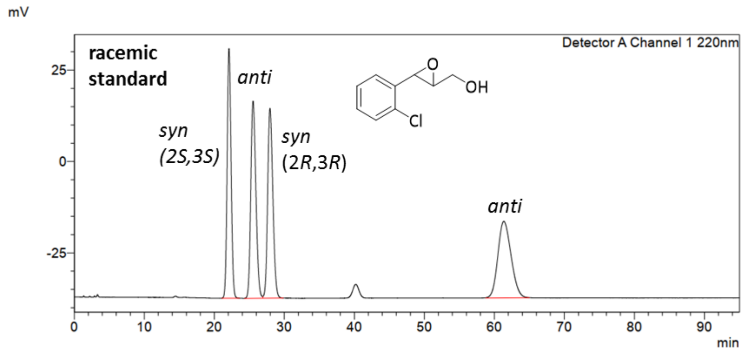

Detector A Channel 1 220nm

Peak#	Ret. Time	Area	Area%
1	22,098	2668339	24,661
2	25,535	2741574	25,338
3	27,952	2674614	24,719
4	61,336	2735490	25,282
Total		10820017	100,000

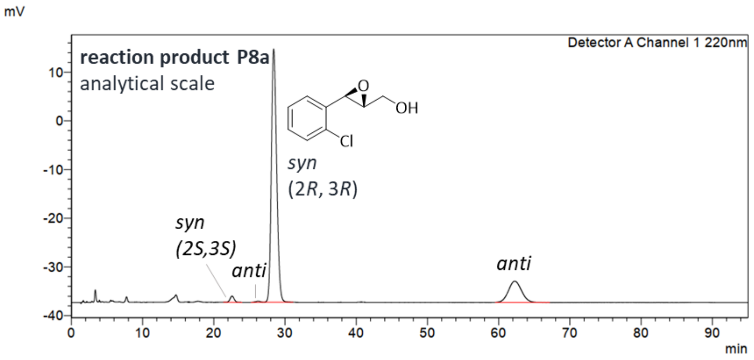

Detector A Channel 1 220nm

Peak#	Ret. Time	Area	Area%
1	22,564	49036	1,486
2	26,234	10060	0,305
3	28,411	2686642	81,416
4	62,253	554152	16,793
Total		3299890	100,000

***e.r.* 98:2**

***d.r.* 83:17**

Figure S7 Chiral HPLC chromatogram of the alcohol derivative of product 3d obtained from enzymatic reaction at analytical scale.

Shown is the racemic standard (top), and the reaction product obtained from conversion by fused 4-OT P8a (bottom).

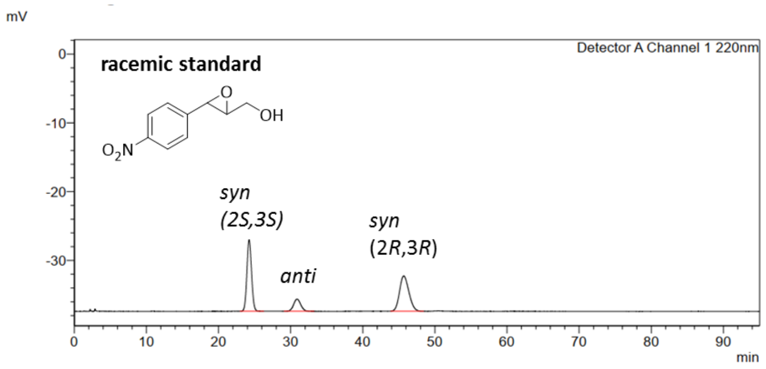

Detector A Channel 1 220nm

Peak#	Ret. Time	Area	Area%
1	24,244	467044	44,412
2	30,928	121078	11,514
3	45,717	463488	44,074
Total		1051609	100,000

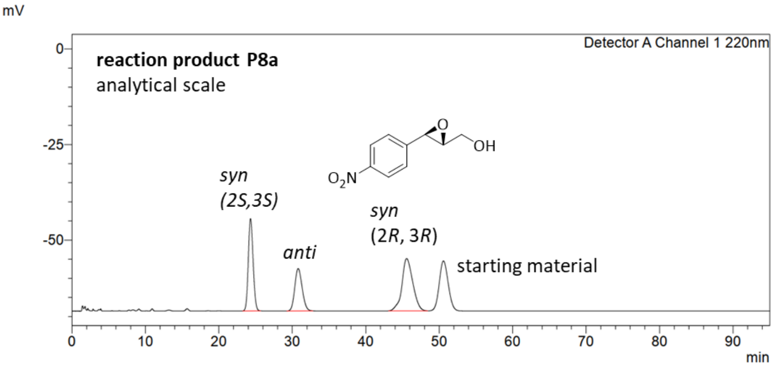

Detector A Channel 1 220nm

Peak#	Ret. Time	Area	Area%
1	24,314	1098045	33,487
2	30,804	777037	23,697
3	45,568	1403977	42,816
Total		3279059	100,000

***e.r.* 56:44**

***d.r.* 76:24**

Figure S8 Chiral HPLC chromatogram of the alcohol derivative of product 3e obtained from enzymatic reaction at analytical scale.

Shown is the racemic standard (top), and the reaction product obtained from conversion by fused 4-OT P8a (bottom).

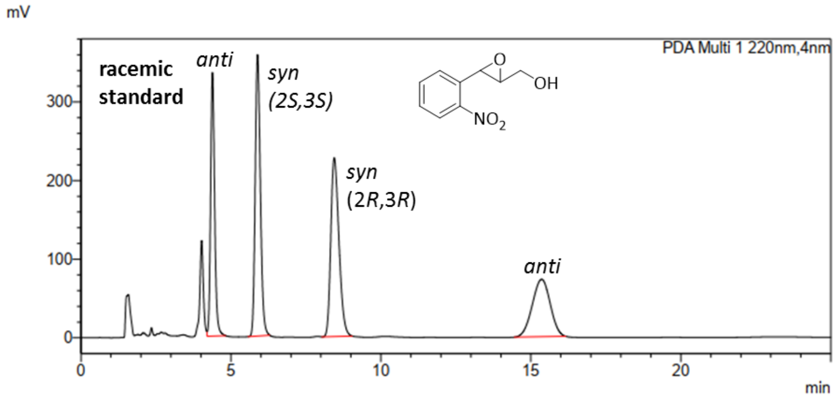

PDA Ch1 220nm

Peak#	Ret. Time	Area	Area%
1	4,382	3137611	21,257
2	5,886	4291343	29,074
3	8,443	4289333	29,060
4	15,355	3041845	20,609
Total		14760133	100,000

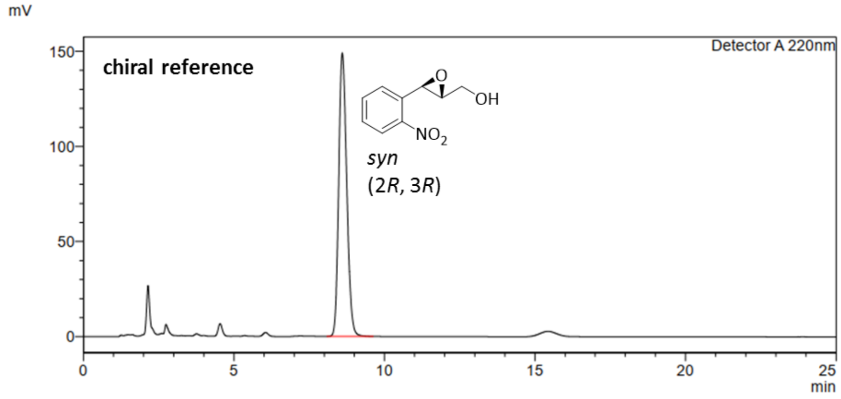

Detector A 220nm

Peak#	Ret. Time	Area	Area%
1	8,599	2753182	100,000
Total		2753182	100,000

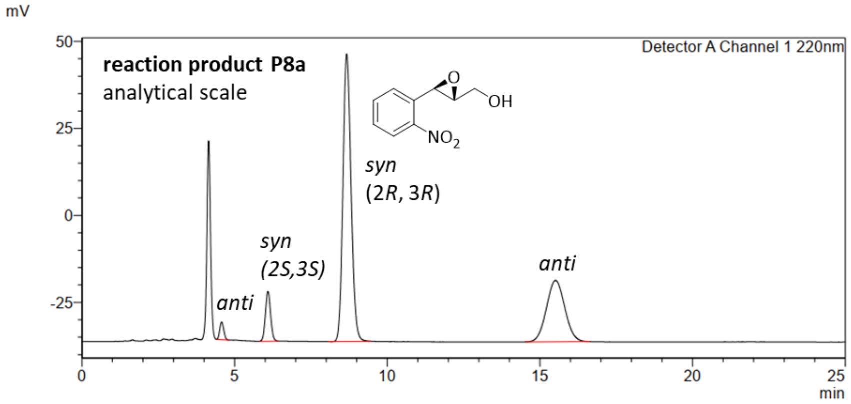

Detector A Channel 1 220nm

Peak#	Ret. Time	Area	Area%
1	4,575	46909	1,895
2	6,092	169929	6,866
3	8,671	1524083	61,579
4	15,513	734082	29,660
Total		2475003	100,000

***e.r.* 90:10**

***d.r.* 68:32**

Figure S9 Chiral HPLC chromatogram of the alcohol derivative of product 3f obtained from enzymatic reaction at analytical scale.

Shown is the racemic standard (top), followed by the chiral reference compound, and the reaction product obtained from conversion by fused 4-OT P8a (bottom).

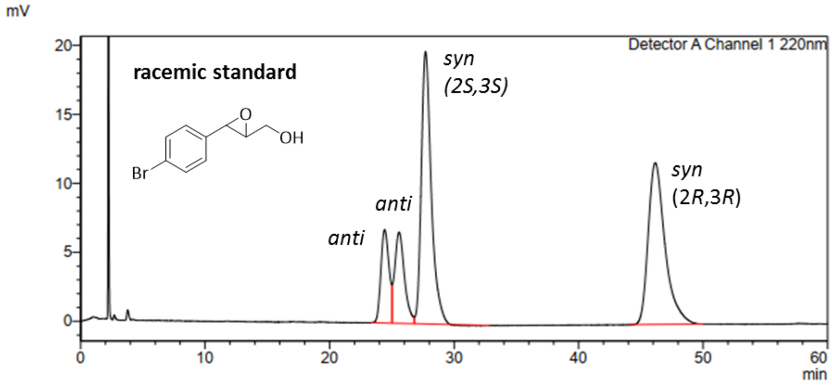

Detector A Channel 1 220nm

Peak#	Ret. Time	Area	Area%
1	24,428	308922	10,778
2	25,581	362043	12,631
3	27,708	1091421	38,077
4	46,155	1103970	38,515
Total		2866357	100,000

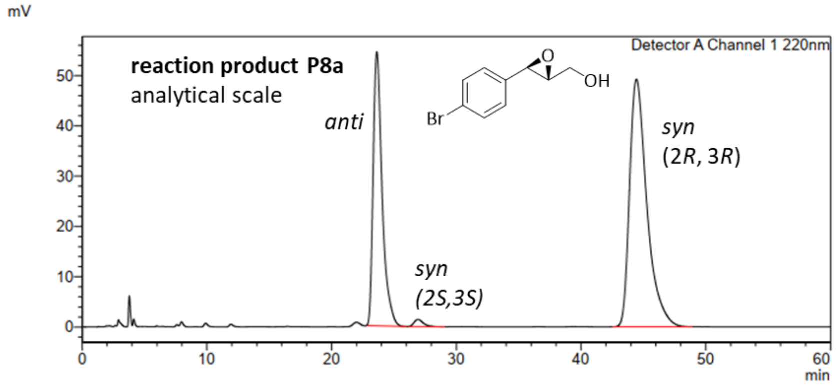

Detector A Channel 1 220nm

Peak#	Ret. Time	Area	Area%
1	23,625	2728072	36,160
2	26,925	72917	0,967
3	44,447	4743403	62,873
Total		7544391	100,000

***e.r.* 98:2**

***d.r.* 64:36**

Figure S10 Chiral HPLC chromatogram of the alcohol derivative of product 3g obtained from enzymatic reaction at analytical scale.

Shown is the racemic standard (top), and the reaction product obtained from conversion by fused 4-OT P8a (bottom).

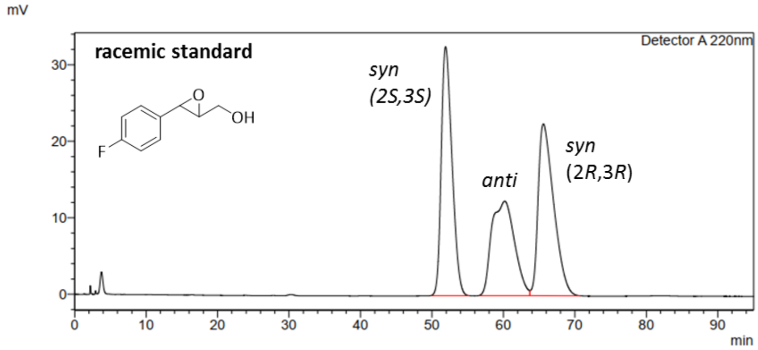

Detector A 220nm

Peak#	Ret. Time	Area	Area%
1	51,904	3389991	35,581
2	60,194	2735526	28,711
3	65,593	3402116	35,708
Total		9527633	100,000

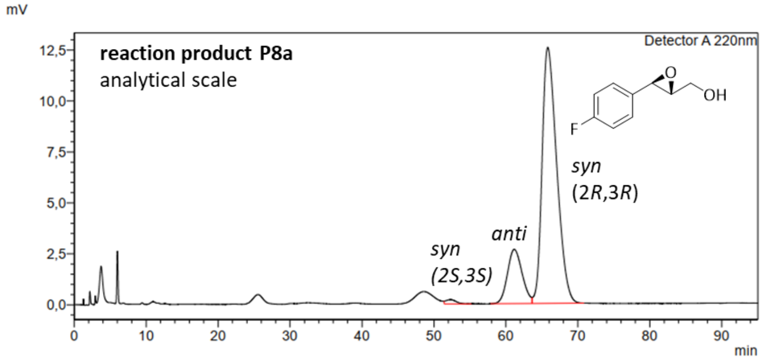

Detector A 220nm

Peak#	Ret. Time	Area	Area%
1	52,370	20913	0,962
2	61,151	379012	17,434
3	65,818	1774056	81,604
Total		2173981	100,000

***e.r.* 99:1**

***d.r.* 83:17**

Figure S11 Chiral HPLC chromatogram of the alcohol derivative of product 3h obtained from enzymatic reaction at analytical scale.

Shown is the racemic standard (top), and the reaction product obtained from conversion by fused 4-OT P8a (bottom).

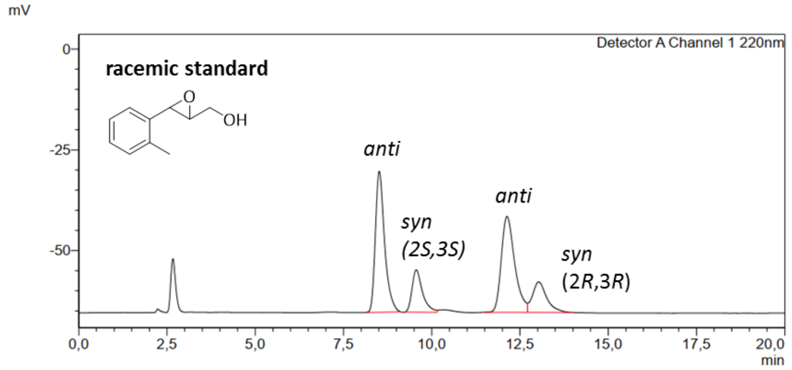

Detector A Channel 1 220nm

Peak#	Ret. Time	Area	Area%
1	8,513	638026	37,519
2	9,562	217567	12,794
3	12,132	631085	37,111
4	13,030	213841	12,575
Total		1700518	100,000

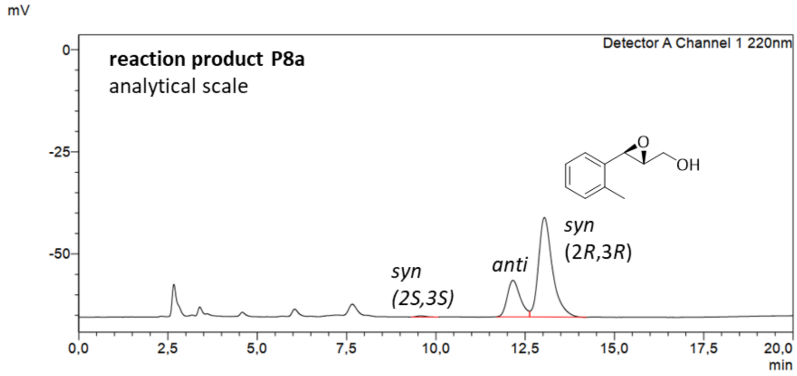

Detector A Channel 1 220nm

Peak#	Ret. Time	Area	Area%
1	9,568	4614	0,514
2	12,158	226419	25,246
3	13,040	665826	74,240
Total		896858	100,000

***e.r.* 99:1**

***d.r.* 75:25**

Figure S3 Chiral HPLC chromatogram of the alcohol derivative of product 3i obtained from enzymatic reaction at analytical scale.

Shown is the racemic standard (top), and the reaction product obtained from conversion by fused 4-OT P8a (bottom).

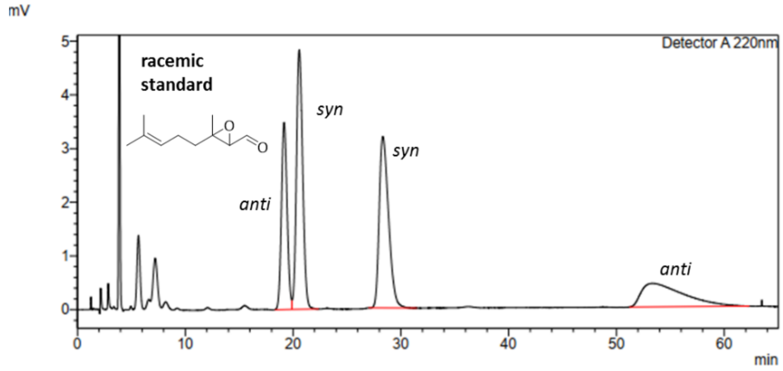

Detector A 220nm

Peak#	Ret. Time	Area	Area%
1	19,171	127919	20,177
2	20,575	193700	30,553
3	28,332	192325	30,337
4	53,339	120026	18,932
Total		633970	100,000

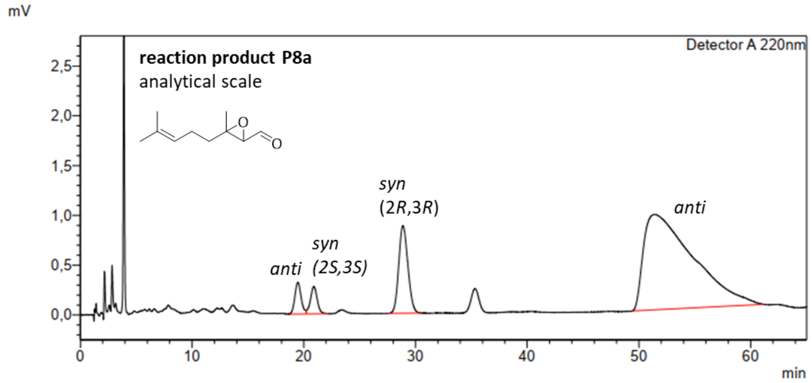

Detector A 220nm

Peak#	Ret. Time	Area	Area%
1	19,483	12469	3,376
2	20,902	11533	3,123
3	28,870	49993	13,537
4	51,383	295300	79,963
Total		369295	100,000

***e.r.* 96:4** (major)

***d.r.* 17:83** (inverted)

Figure S4 Chiral HPLC chromatogram of the alcohol derivative of product 3j obtained from enzymatic reaction at analytical scale.

Shown is the racemic standard (top), and the reaction product obtained from conversion by fused 4-OT P8a (bottom).

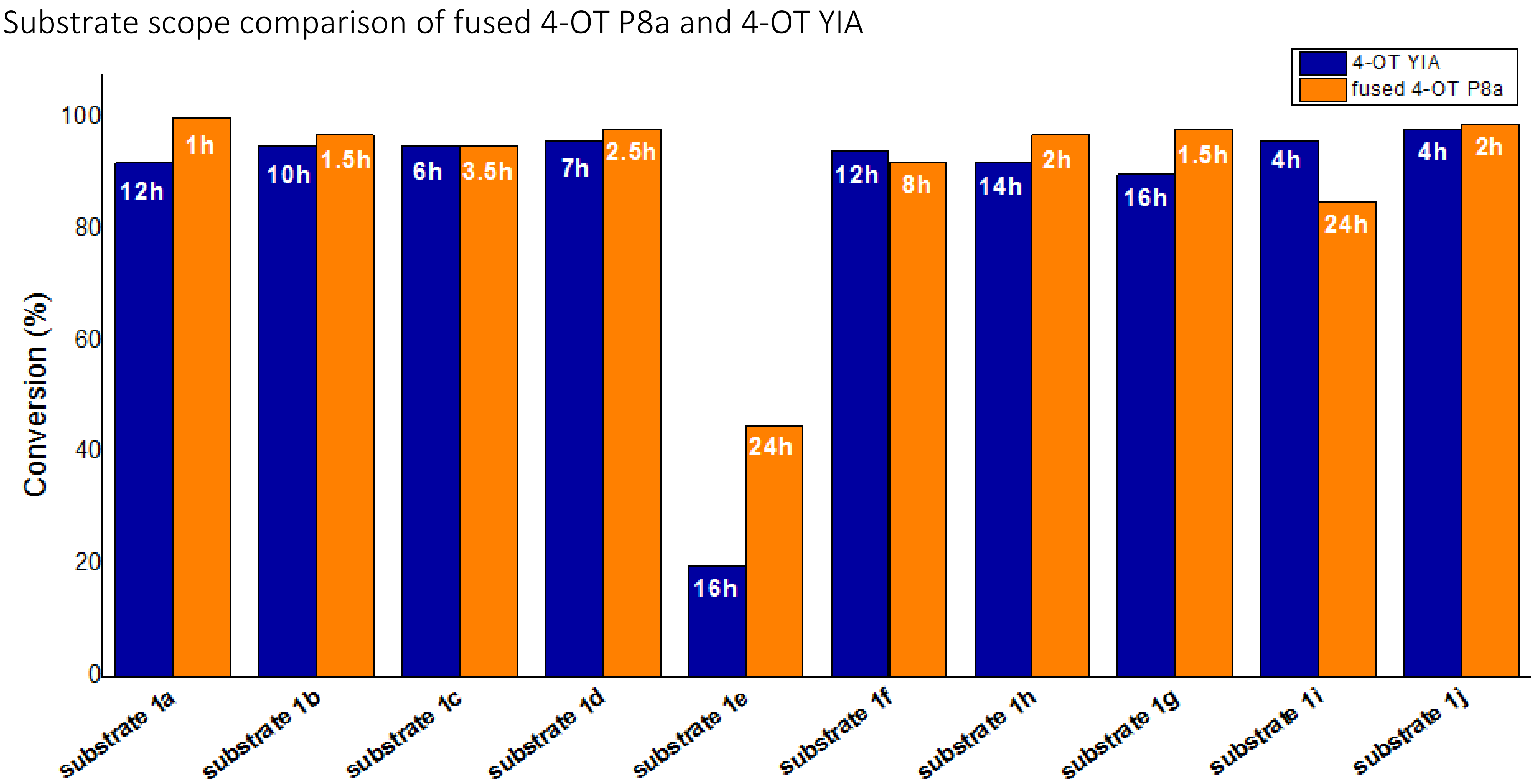

Figure S14 Comparison of the conversion of different cinnamaldehyde derivatives catalysed by 4-OT YIA or fused 4-OT P8a with H_2O_2. The reaction was conducted in 20 mM sodium phosphate buffer (pH 6.3) with 1 mM of aldehyde, 10 µM fused 4-OT P8a or 4-OT YIA and 50 mM H_2O_2 (P8a) or 25 mM H_2O_2 (YIA). The conversion (%) was determined by GC-MS analysis. The duration of the conversion is given in hours. The data of the GC-MS analysis for 4-OT YIA was obtained from Xu *et al.*, 2020[2].

Table S4 Comparison of the enantiomeric and diastereomeric ratio of the epoxide product after the peroxygenation of different cinnamaldehyde derivatives with H_2O_2 catalysed by 4-OT YIA or fused 4-OT P8a.

The reaction was conducted in 20 mM sodium phosphate buffer (pH 6.3) with 1 mM of aldehyde, 10 µM enzyme and 50 mM H_2O_2 (P8a) or 25 mM H_2O_2 (YIA). Enantiomeric and diastereomeric ratios were determined by chiral HPLC analysis. The data of the chiral HPLC analysis of 4-OT YIA was retrieved from Xu *et al.*, 2020.[2] n.d. = not determined.

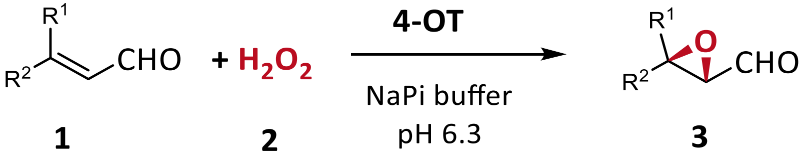

substrate	R^1	R^2	product	*e.r.* fused 4-OT P8a	*e.r.* 4-OT YIA	*d.r.* fused 4-OT P8a	*d.r.* 4-OT YIA	Abs. config.
**1a** cinnamaldehyde	H	Ph	**3a**	99:1	92:8	96:4	84:16	(2*S*,3*R*)
**1b** *p*-chlorocinnamaldehyde	H	*p*-Cl-Ph	**3b**	99:1	76:24	70:30	74:26	(2*S*,3*R*)
**1c** *m*-chlorocinnamaldehyde	H	*m*-Cl-Ph	**3c**	98:2	74:26	80:20	85:15	(2*S*,3*R*)
**1d** *o*-chlorocinnamaldehyde	H	*o*-Cl-Ph	**3d**	98:2	88:12	83:17	80:20	(2*S*,3*R*)
**1e** *p*-nitrocinnamaldehyde	H	*p*-NO_2-Ph	**3e**	56:44	n.d.	76:24	n.d.	(2*S*, 3*R*)
**1f** *o*-nitrocinnamaldehyde	H	*o*-NO_2-Ph	**3f**	90:10	84:16	68:32	82:18	(2*S*,3*R*)
**1g** *p*-bromocinnamaldehyde	H	*p*-Br-Ph	**3g**	98:2	92:8	64:36	84:16	(2*S*,3*R*)
**1h** *p*-fluorocinnamaldehyde	H	*p*-F-Ph	**3h**	99:1	89:11	83:17	77:23	(2*S*,3*R*)
**1i** *o*-methylcinnamaldehyde	H	o-Me-Ph	**3i**	99:1	94:6	75:25	68:32	(2*S*,3*R*)
**1j** citral	Me	$(CH_3)_2CCHCH_2CH_2$	**3j**	96:4	95:5	17:83	20:80	n.d.

Comparison of the peroxygenase activity of different 4-OT variants using either hydrogen peroxide or tert-butyl peroxide as nucleophile

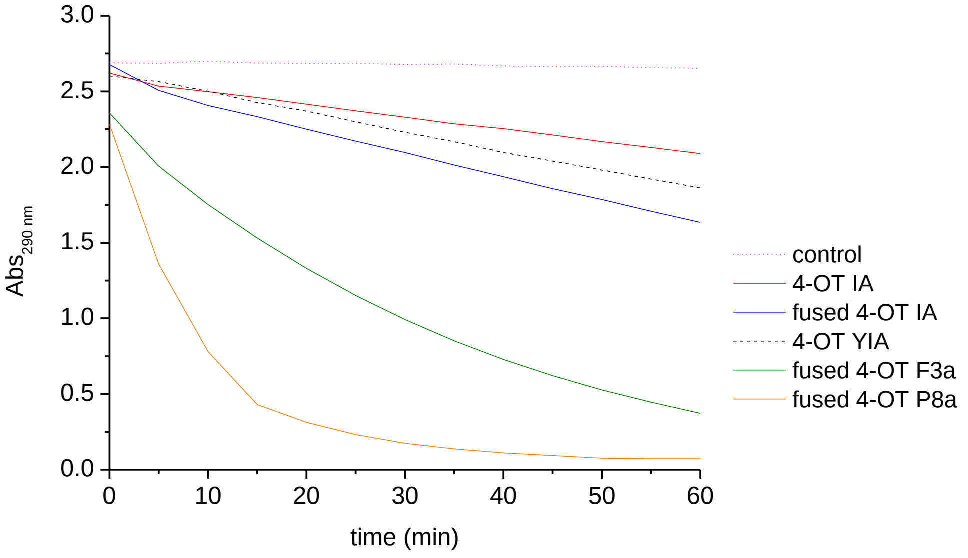

A

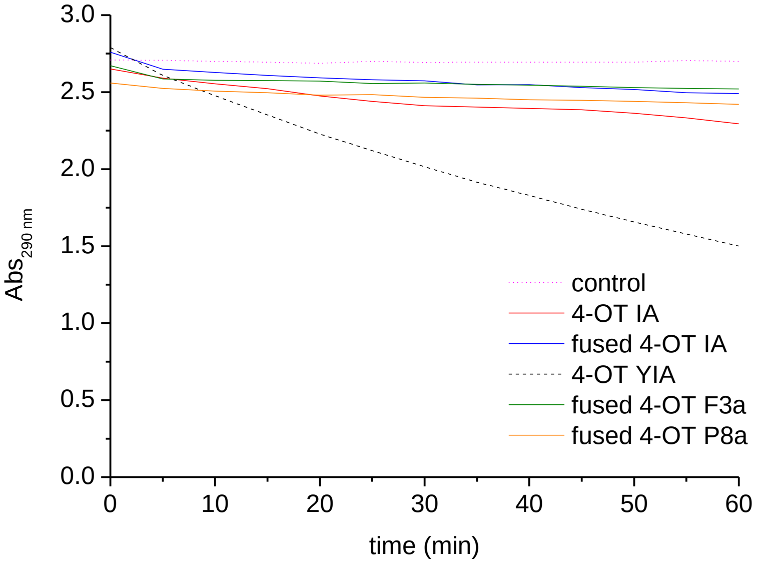

B

Figure S15 Comparison of the cinnamaldehyde depletion representing the epoxidation activity of different 4-OT variants applying hydrogen peroxide or tert-butyl peroxide as oxidant.

The reaction was conducted in 20 mM sodium phosphate buffer (pH 6.3) with 1 mM cinnamaldehyde, 50 mM H_2O_2 (**A**) or 200 mM tert-butyl peroxide (**B**) and 10 µM of enzyme. Cinnamaldehyde depletion was followed at 290 nm over 60 min. As a control, the reaction without enzyme was followed.

Comparison of the enantioselectivity of different 4-OT mutants catalysing epoxidation reactions at semi-preparative scale

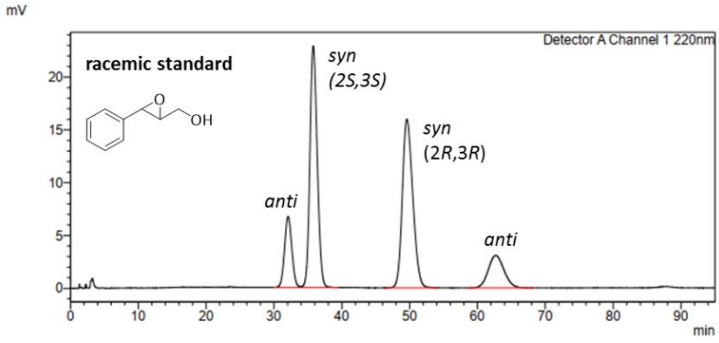

Peak#	Ret. Time	Area	Area%
1	32,071	502042	11,333
2	35,781	1716684	38,751
3	49,591	1713477	38,678
4	62,614	497885	11,239
Total		4430088	100,000

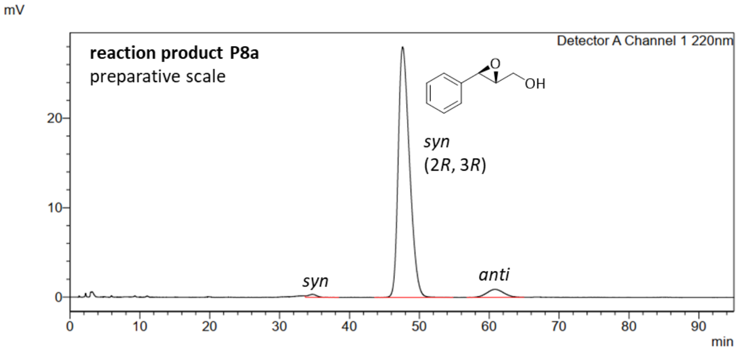

Peak#	Ret. Time	Area	Area%
1	34,683	28389	0,855
2	47,609	3141244	94,626
3	60,751	150012	4,519
Total		3319645	100,000

***e.r.* 99:1**

***d.r.* 95:5**

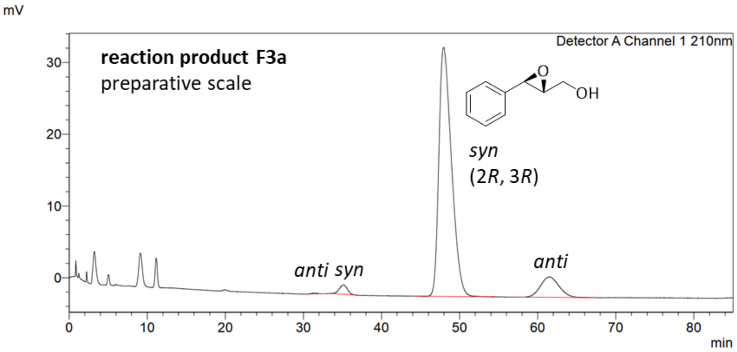

Peak#	Ret. Time	Area	Area%
1	31,446	6635	0,148
2	35,091	95367	2,134
3	47,947	3894939	87,155
4	61,483	472047	10,563
Total		4468987	100,000

***e.r.* 98:2**

***d.r.* 89:11**

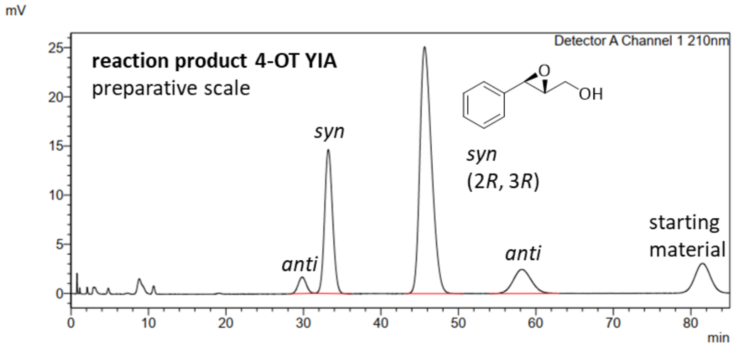

Peak#	Ret. Time	Area	Area%
1	29,863	122015	2,896
2	33,206	1048221	24,883
3	45,634	2643551	62,753
4	58,229	398857	9,468
Total		4212644	100,000

***e.r.* 72:28**

***d.r.* 88:12**

Figure S16 Chiral HPLC analysis of product 3a obtained from enzymatic conversions at semi-preparative scale catalysed by fused 4-OT P8a or F3a, or 4-OT YIA.

From the top to the bottom, the graphs show as follows: racemic standard and the reaction products obtained from conversion catalysed by fused 4-OT P8a, F3a, or 4-OT YIA. To compare the enantioselectivity under preparative-scale conditions, a reaction mixture was set up in a final volume of 1 mL containing 10 mM cinnamaldehyde derivative, 50 mM H_2O_2, 5% (v/v) ethanol and 100 µM of enzyme in 20 mM sodium phosphate buffer (pH 6.3).

Preparative-scale synthesis applying fused 4-OT P8a

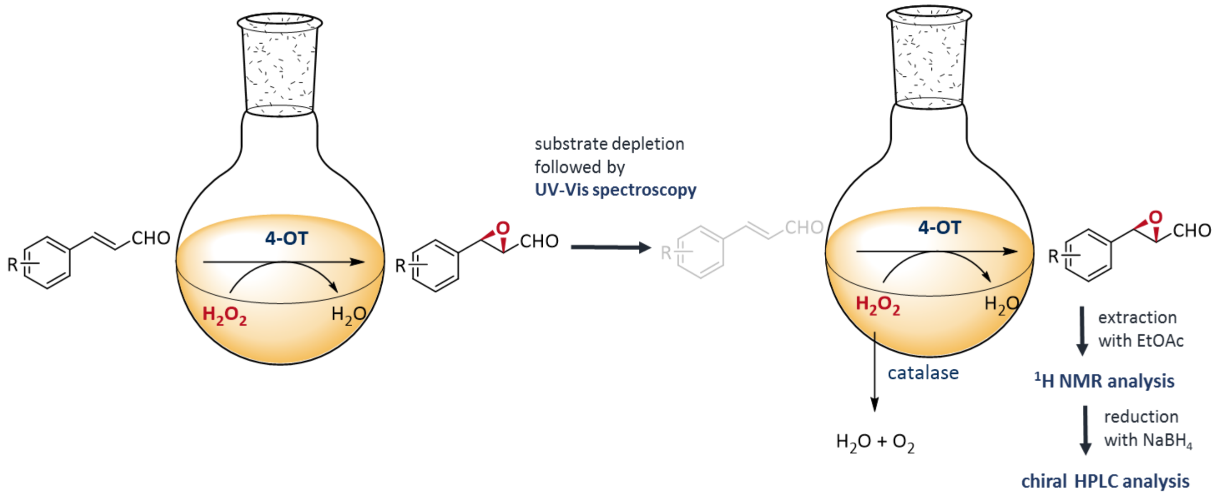

Figure S17 Schematic overview of the preparative-scale synthesis of epoxides from cinnamaldehyde derivatives and hydrogen peroxide applying fused 4-OT P8a.

Product 3a

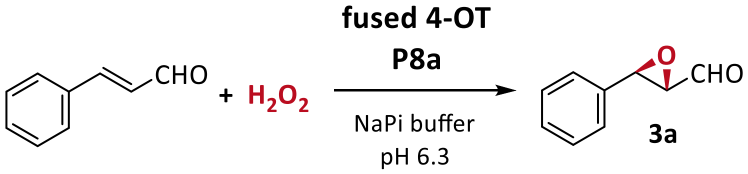

time 2 h
conversion >99%
yield 76% (40 mg)
e.r. 99:1; d.r. 96:4
abs. config. 2*S*,3*R*

A

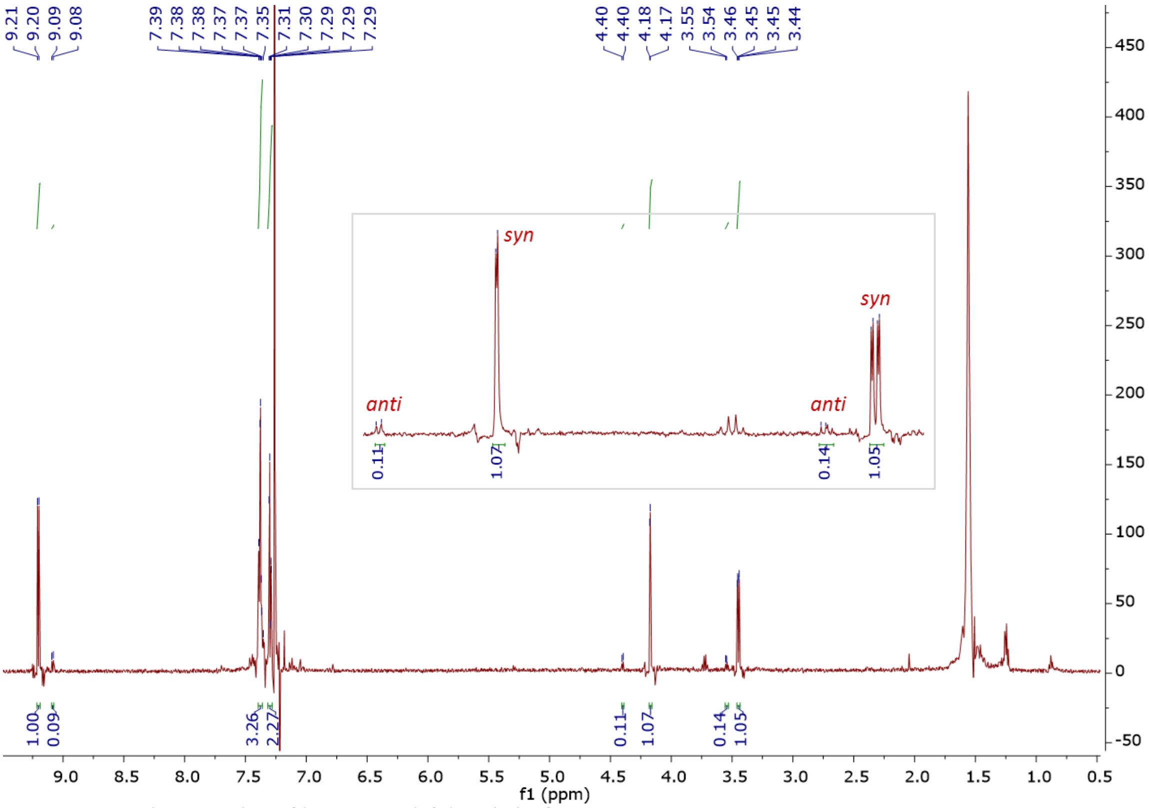

B

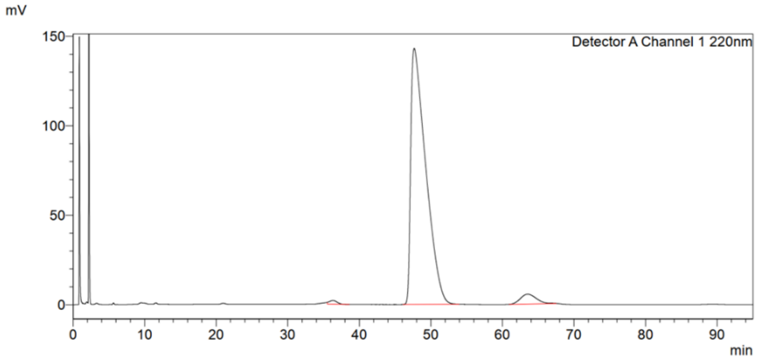

Detector A Channel 1 220nm

Peak#	Ret. Time	Area	Area%
1	36,347	158331	0,724
2	47,672	20866992	95,404
3	63,532	846845	3,872
Total		21872168	100,000

C

Figure S18 ^{1}H NMR analysis and chiral HPLC chromatogram from preparative-scale synthesis of product 3a applying fused 4-OT P8a.

Overview of the reaction showing the yield of the crude product (**A**), ^{1}H NMR spectrum in $CDCl_3$ of the crude product (**B**), and chiral HPLC chromatogram of the reduced crude product (**C**). (2S,3R)-3-phenyloxirane-2-carbaldehyde, yellow oil, ^{1}H NMR (500 MHz, $CDCl_3$, major diastereomer): δ (ppm) 9.20 (d, J = 6.1 Hz, 1H), 7.39–7.29 (m, 5H), 4.17 (d, J = 1.7 Hz, 1H), 3.45 (dd, J = 1.8, 6.1 Hz, 1H). ^{1}H NMR data is in agreement with literature.[3]

Product **3c**

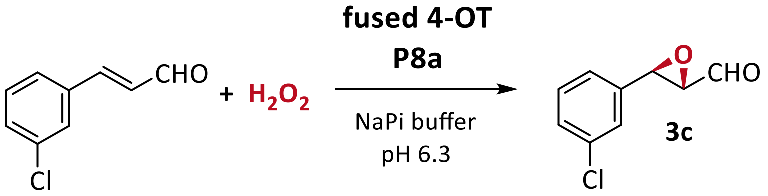

time 4.5 h
conversion >99%
yield 75% (25 mg)
e.r. 97:3; d.r. 87:13
abs. config. 2*S*,3*R*

A

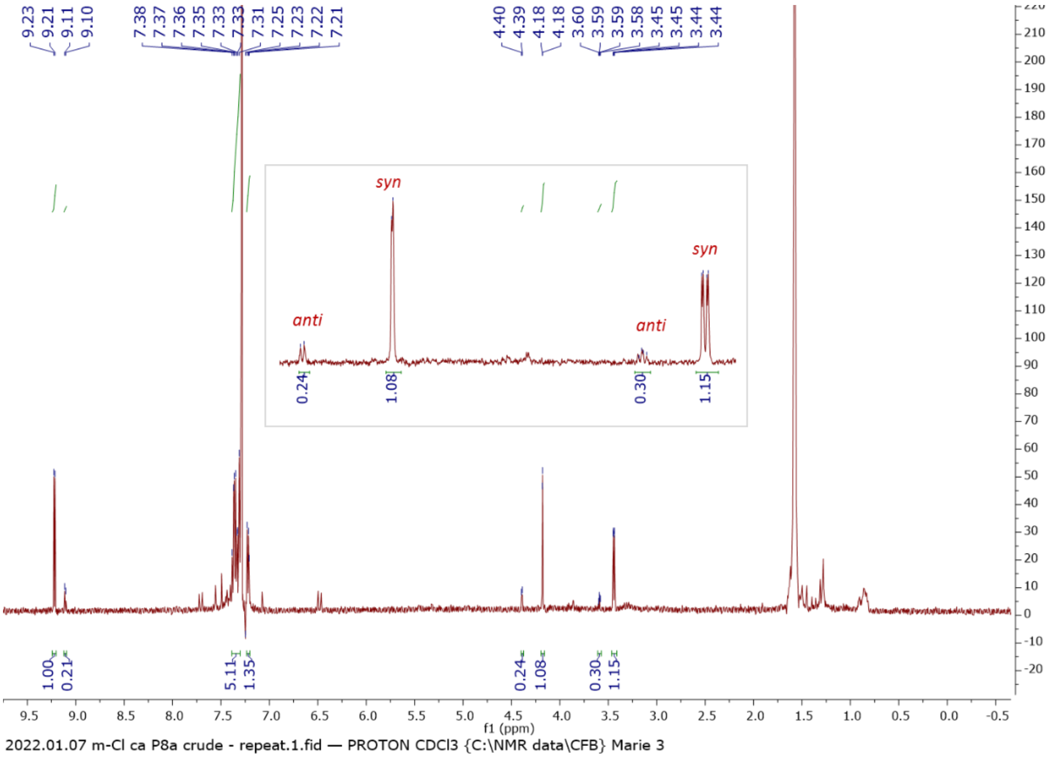

B

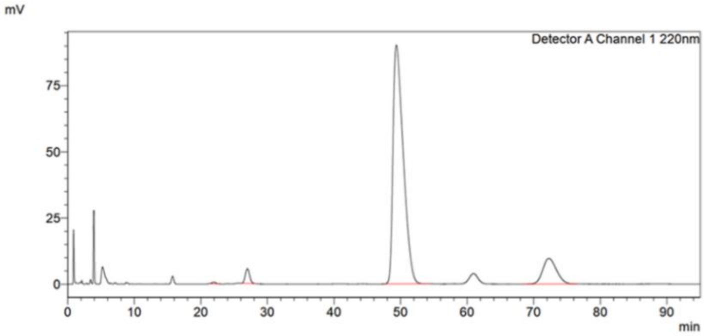

Detector A Channel 1 220nm

Peak#	Ret. Time	Area	Area%
1	21,972	25609	0,226
2	27,000	252435	2,232
3	49,369	9640174	85,244
4	72,262	1390644	12,297
Total		11308861	100,000

C

Figure S19 ^{1}H NMR analysis and chiral HPLC chromatogram from preparative-scale synthesis of product 3c applying fused 4-OT P8a.

Overview of the reaction showing the yield of the crude product (**A**), ^{1}H NMR spectrum in $CDCl_3$ of the crude product (**B**), and chiral HPLC chromatogram of the reduced crude product (**C**). (2*S*,3*R*)-3-(3-chlorophenyl)oxirane-2-carbaldehyde, yellow oil, ^{1}H NMR (500 MHz, $CDCl_3$, major diastereomer): δ (ppm) 9.22 (d, J = 6.02 Hz, 1H), 7.40 – 7.19 (m, 5H), 4.18 (d, J = 1.53 Hz, 1H), 3.44 (dd, J = 1.72, 6.00 Hz, 1H). ^{1}H NMR data of the corresponding alcohol: ^{1}H NMR (500 MHz, $CDCl_3$) δ (ppm) 7.32 – 7.14 (m, 5H), 4.05 (d, J = 14.0 Hz, 1H), 3.92 (d, J = 1.9 Hz, 1H), 3.83 (dd, J = 12.1, 4.1 Hz, 1H), 3.18 (q, J = 2.7 Hz, 1H), 2.05 (s, 1H) is in agreement with literature.[2]

Product **3d**

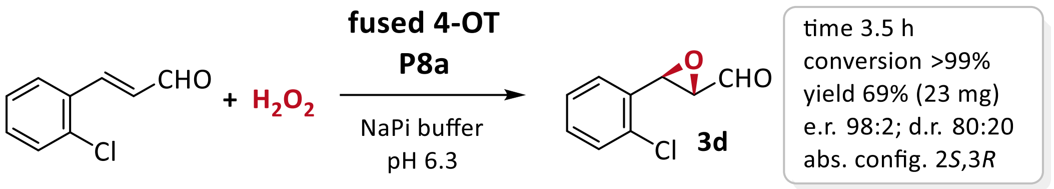

A

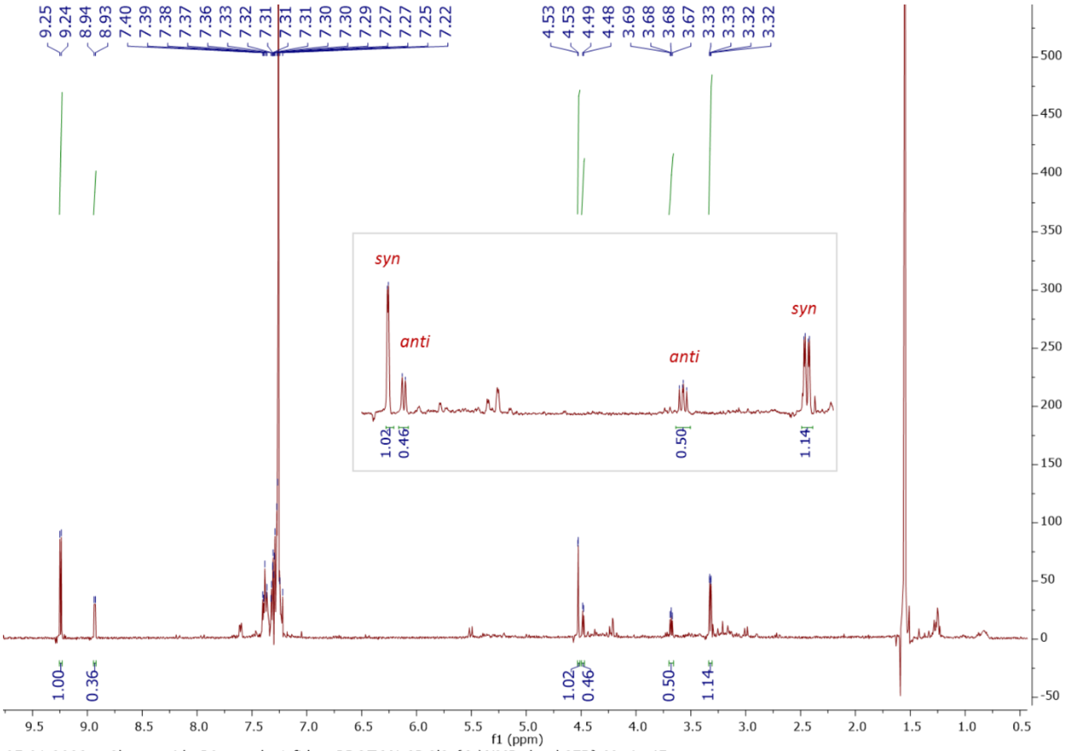

05.01.2022 o-Clcaepoxide P8a crude.1.fid — PROTON CDCl3 {C:\NMR data\CFB} Marie 45

B

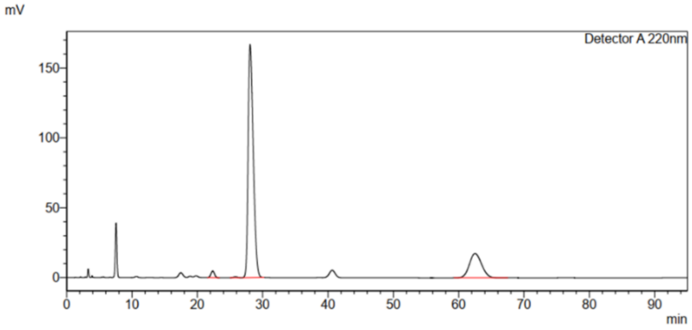

Detector A 220nm

Peak#	Ret. Time	Area	Area%
1	22,352	184195	1,595
2	25,832	30853	0,267
3	28,078	9060822	78,467
4	62,511	2271464	19,671
Total		11547334	100,000

C

Figure S20 ^{1}H NMR analysis and chiral HPLC chromatogram from preparative-scale synthesis of product 3d applying fused 4-OT P8a.

Overview of the reaction showing the yield of the crude product (**A**), ^{1}H NMR spectrum in $CDCl_3$ of the crude product (**B**), and chiral HPLC chromatogram of the reduced crude product (**C**). (2*S*,3*R*)-3-(2-chlorophenyl)oxirane-2-carbaldehyde, yellow oil, ^{1}H NMR (500 MHz, $CDCl_3$, major diastereomer): δ (ppm) 9.24 (d, J = 6.09 Hz, 1H), 7.40 – 7.22 (m, 5H), 4.53 (d, J = 1.67 Hz, 1H), 3.32 (dd, J = 1.81, 6.08 Hz, 1H). ^{1}H NMR data of the corresponding alcohol: ^{1}H NMR (500 MHz, $CDCl_3$) δ (ppm) 7.38 – 7.34 (m, 2H), 7.30 – 7.26 (m, 3H), 4.25 (d, J = 2.0 Hz, 1H), 4.10 – 4.06 (m, 1H), 3.85 (ddd, J = 12.2, 7.6, 4.0 Hz, 1H), 3.10 (dt, J = 4.3, 2.3 Hz, 1H), 1.72 (dd, J = 7.6, 5.5 Hz, 1H) is in agreement with literature.[2]

Product **3g**

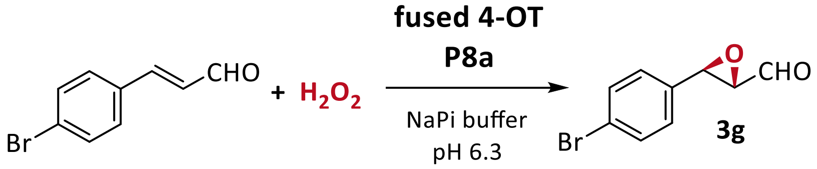

time 3 h
conversion >99%
yield 78% (33 mg)
e.r. 96:4; d.r. 67:33
abs. config. 2*S*,3*R*

A

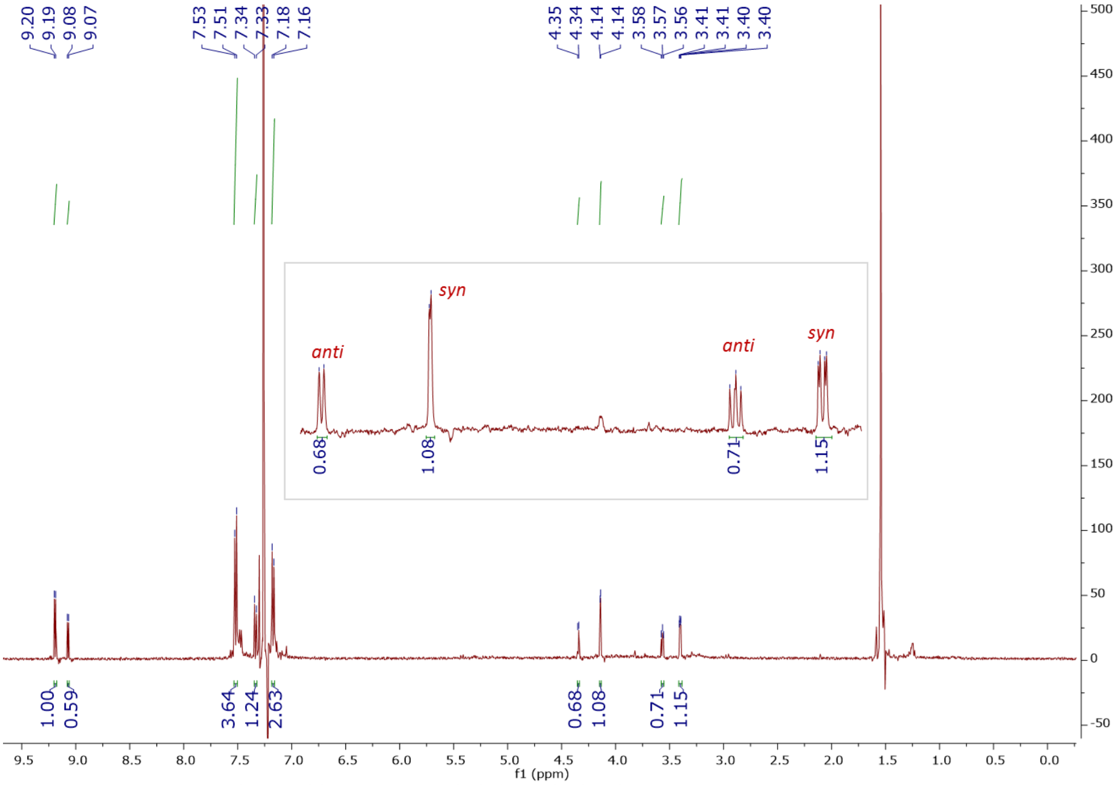

MCS 16.12.2021 p-Br-caepoxide F8a.1.fid — PROTON CDCl3 {C:\NMR data\CFB} Marie 7

B

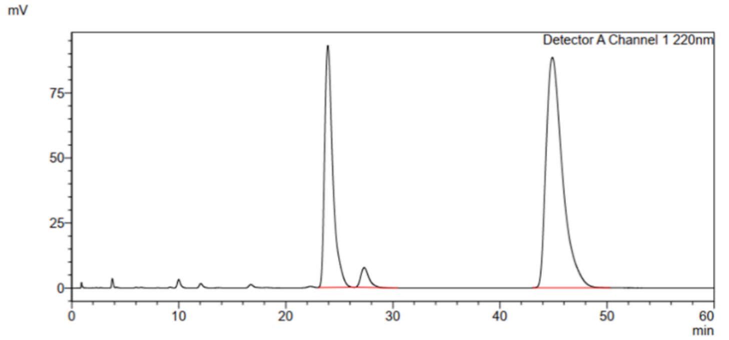

Detector A Channel 1 220nm

Peak#	Ret. Time	Area	Area%
1	23,929	4937017	33,457
2	27,320	400203	2,712
3	44,897	9419195	63,831
Total		14756414	100,000

C

Figure S21 ^{1}H NMR analysis and chiral HPLC chromatogram from preparative-scale synthesis of product 3g applying fused 4-OT P8a.

Overview of the reaction showing the yield of the crude product (**A**), ^{1}H NMR spectrum in $CDCl_3$ of the crude product (**B**), and chiral HPLC chromatogram of the reduced crude product (**C**). (2*S*,3*R*)-3-(4-bromophenyl)oxirane-2-carbaldehyde, colourless oil, ^{1}H NMR (500 MHz, $CDCl_3$, major diastereomer): δ (ppm) 9.19 (d, J = 5.98 Hz, 1H), 7.53 – 7.50 (d, J = 8.43 Hz, 4H), 7.35 – 7.33 (d, J = 8.58 Hz, 1H), 7.18 – 7.16 (d, J = 8.58 Hz, 3H), 4.14 (d, J = 1.58 Hz, 1H), 3.40 (dd, J = 1.73, 6.03 Hz, 1H). The data is in agreement with literature.[4]

Product 3h

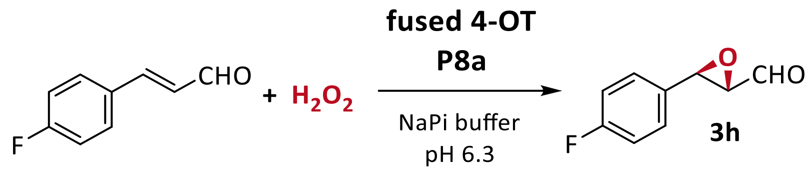

time 3 h
conversion >99%
yield 67% (40 mg)
e.r. 98:2; d.r. 87:13
abs. config. 2*S*,3*R*

A

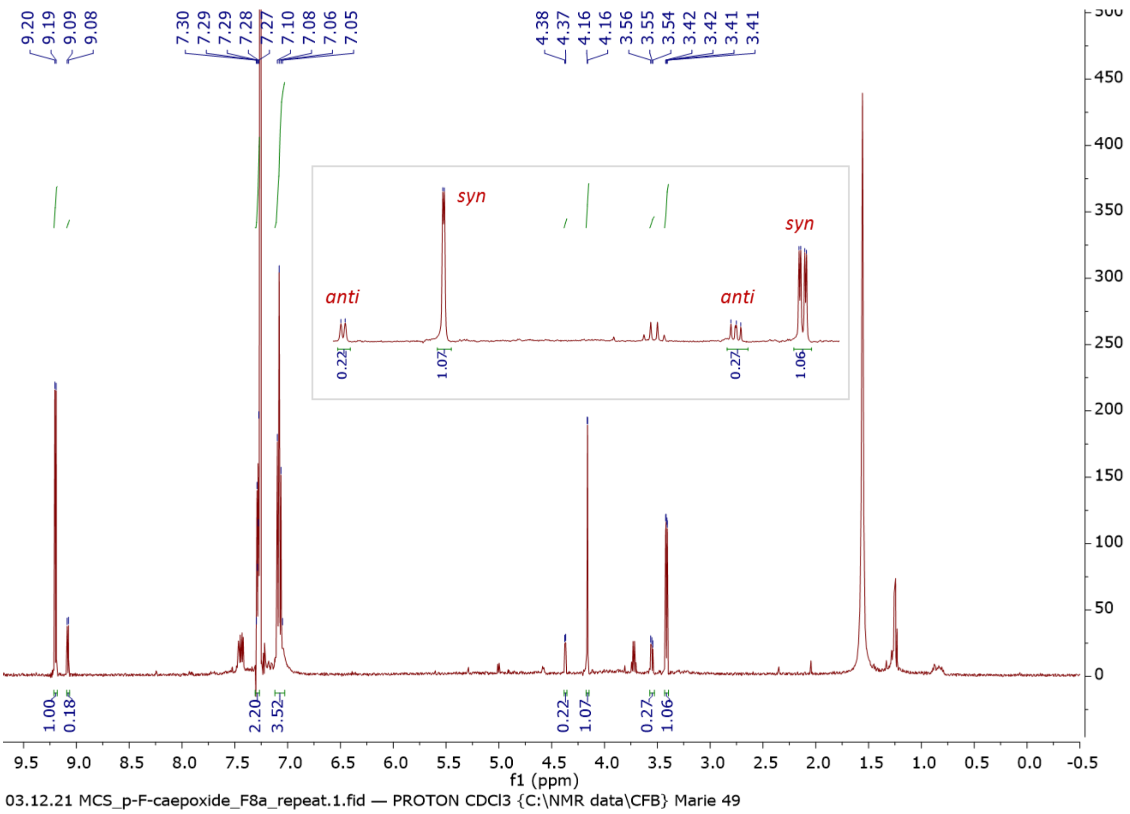

B

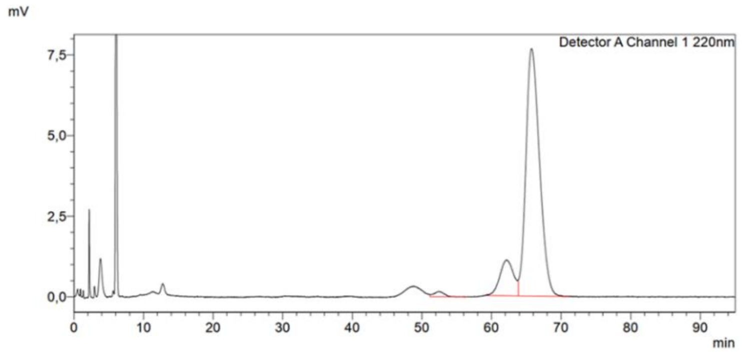

Detector A Channel 1 220nm

Peak#	Ret. Time	Area	Area%
1	52,621	16514	1,361
2	62,153	153073	12,614
3	65,781	1043954	86,025
Total		1213542	100,000

C

Figure S22 ^{1}H NMR analysis and chiral HPLC chromatogram from preparative-scale synthesis of product 3h applying fused 4-OT P8a.

Overview of the reaction showing the yield of the crude product (**A**), ^{1}H NMR spectrum in $CDCl_3$ of the crude product (**B**), and chiral HPLC chromatogram of the reduced crude product (**C**). (2*S*,3*R*)-3-(4-fluorophenyl)oxirane-2-carbaldehyde, yellow oil, ^{1}H NMR (500 MHz, $CDCl_3$, major diastereomer): δ (ppm) 9.20 (d, J = 6.01 Hz, 1H), 7.32 – 7.21 (m, 2H) 7.12 – 7.01 (m, 3H), 4.16 (d, J = 1.68 Hz, 1H), 3.41 (dd, J = 1.75, 6.00 Hz, 1H). The data is in agreement with literature.[4]

Gram-scale synthesis of product **3a**

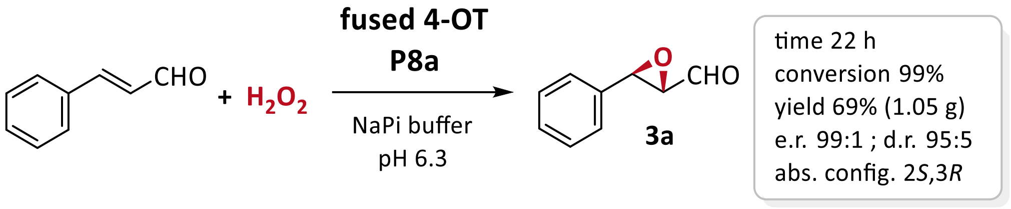

A

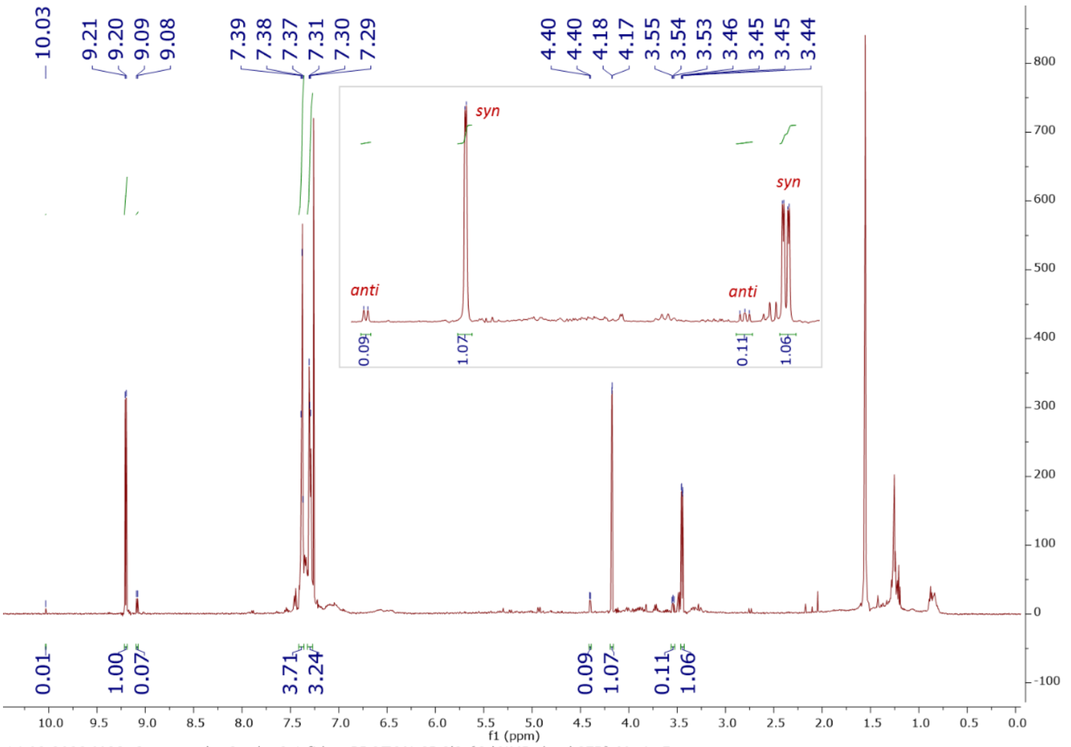

14.02.2022 MCS_Gram_scale_Crude_3.1.fid — PROTON CDCl3 {C:\NMR data\CFB} Marie 5

B

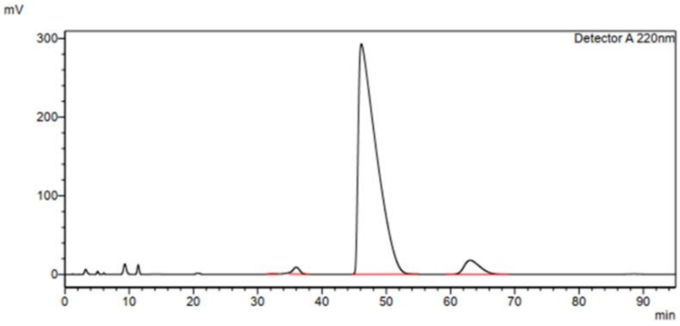

Detector A 220nm

Peak#	Ret. Time	Area	Area%
1	32,360	28743	0,047
2	35,979	666201	1,097
3	46,119	56929279	93,758
4	63,052	3094930	5,097
Total		60719154	100,000

C

Figure S23 ^{1}H NMR analysis and chiral HPLC chromatogram from gram-scale synthesis of product 3a applying fused 4-OT P8a.

Overview of the reaction showing the yield of the crude product (**A**), ^{1}H NMR spectrum in $CDCl_3$ of the crude product (**B**), and chiral HPLC chromatogram of the reduced crude product (**C**). (2S,3R)-3-phenyloxirane-2-carbaldehyde, yellow oil, ^{1}H NMR (500 MHz, $CDCl_3$, major diastereomer): δ (ppm) 9.20 (d, J = 6.1 Hz, 1H), 7.39–7.29 (m, 5H), 4.17 (d, J = 1.7 Hz, 1H), 3.45 (dd, J = 1.8, 6.1 Hz, 1H). ^{1}H NMR data is in agreement with literature.[3]

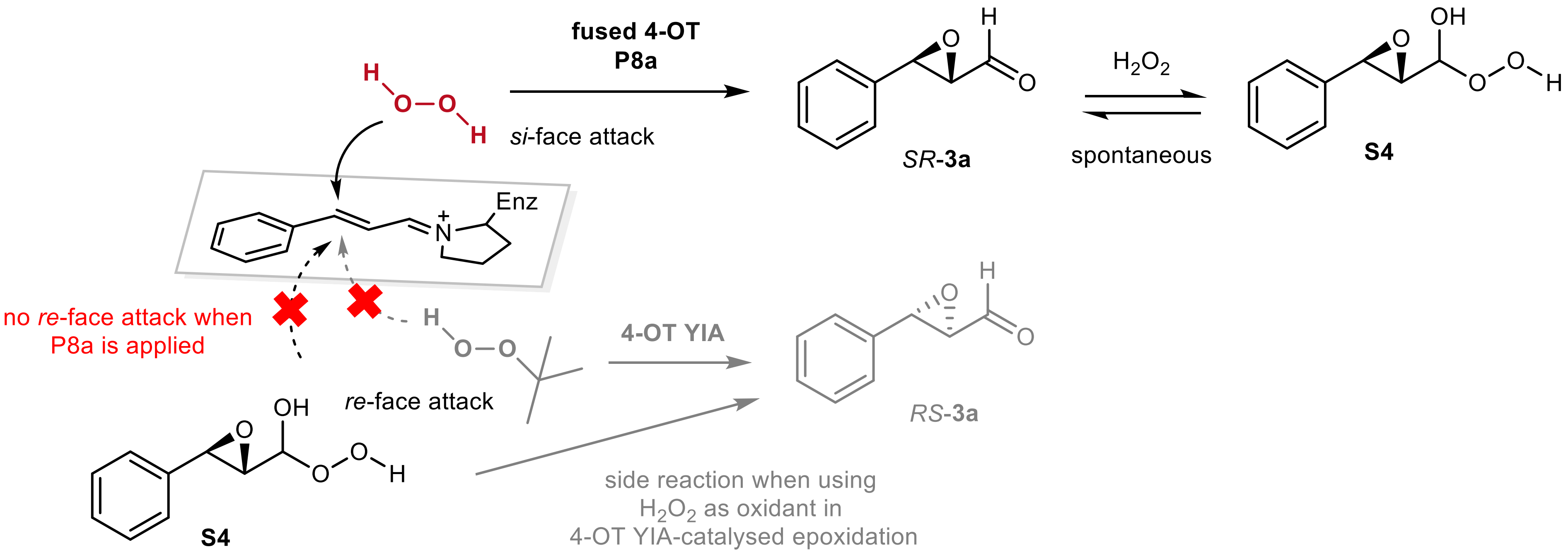

Figure S24 Proposed mechanism of preventing the side reaction and reduction in enantioselectivity in the fused 4-OT P8a-catalyzed epoxidation using H_2O_2 as oxidant.

Modified figure from Xu *et al.*, 2020[2].

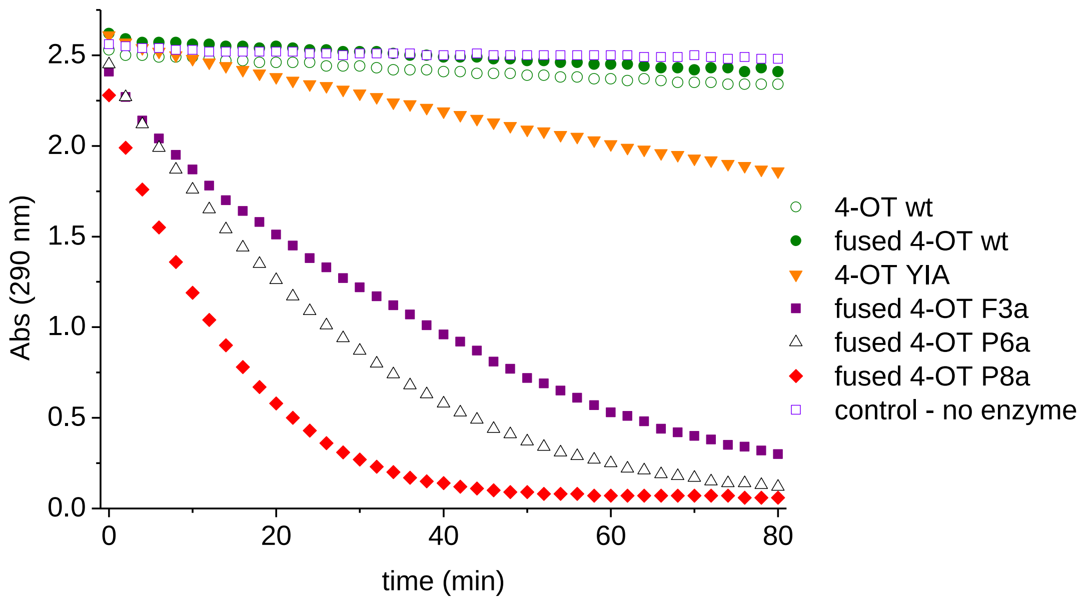

Figure S25 Comparison of the peroxygenase activity of 4-OT wild type, fused 4-OT wild type and engineered 4-OT variants.
Reaction progress curves following the depletion of 1 mM **1a** with 50 mM H_2O_2 in the presence of 10 µM enzyme and 5% (*v*/*v*) ethanol in 20 mM sodium phosphate buffer at pH 6.3.

Supporting references

[1] G. Xu, A. Kunzendorf, M. Crotti, H. J. Rozeboom, A. M. W. H. Thunnissen, G. J. Poelarends, *Angew. Chem. Int. Ed.* **2022**, DOI 10.1002/anie.202113970.

[2] G. Xu, M. Crotti, T. Saravanan, K. M. Kataja, G. J. Poelarends, *Angew. Chem. Int. Ed.* **2020**, *59*, 10374–10378.

[3] M. Marigo, J. Franzén, T. B. Poulsen, W. Zhuang, K. A. Jørgensen, *J. Am. Chem. Soc.* **2005**, *127*, 6964–6965.

[4] C. Sparr, W. B. Schweizer, H. M. Senn, R. Gilmour, *Angew. Chem. Int. Ed.* **2009**, *48*, 3065–3068.
